# PIM1 kinase promotes EMT-associated osimertinib resistance via regulating GSK3β signaling pathway in EGFR-mutant non-small cell lung cancer

**DOI:** 10.1038/s41419-024-07039-0

**Published:** 2024-09-03

**Authors:** Jing Zhou, Xinyue Wang, Zhaona Li, Fan Wang, Lianjing Cao, Xiuqiong Chen, Dingzhi Huang, Richeng Jiang

**Affiliations:** 1 Tianjin Medical University Cancer Institute and Hospital, National Clinical Research Center for Cancer, Key Laboratory of Cancer Prevention and Therapy, Tianjin’s Clinical Research Center for Cancer, Tianjin, China; 2 Department of Thoracic Oncology, Tianjin Lung Cancer Center, Tianjin Cancer Institute and Hospital, Tianjin Medical University, Tianjin, China; 3 Department of Oncology, Qilu Hospital of Shandong University Dezhou Hospital, Dezhou, China; 4 The affiliated Huaian No.1 People’s Hospital of Nanjing Medical University, Huaian, China; 5 Department of Radiation Oncology, The Affiliated Hospital of Qingdao University, Qingdao, China; 6 Department of Cancer Center, Daping Hospital, Army Medical University, Chongqing, China; 7 Tianjin Cancer Hospital Airport Hospital, National Clinical Research Center for Cancer, Tianjin, China

**Keywords:** Cancer therapeutic resistance, Non-small-cell lung cancer

## Abstract

Acquired resistance is inevitable in the treatment of non-small cell lung cancer (NSCLC) with osimertinib, and one of the primary mechanisms responsible for this resistance is the epithelial-mesenchymal transition (EMT). We identify upregulation of the proviral integration site for Moloney murine leukemia virus 1 (PIM1) and functional inactivation of glycogen synthase kinase 3β (GSK3β) as drivers of EMT-associated osimertinib resistance. Upregulation of PIM1 promotes the growth, invasion, and resistance of osimertinib-resistant cells and is significantly correlated with EMT molecules expression. Functionally, PIM1 suppresses the ubiquitin-proteasome degradation of snail family transcriptional repressor 1 (SNAIL) and snail family transcriptional repressor 2 (SLUG) by deactivating GSK3β through phosphorylation. The stability and accumulation of SNAIL and SLUG facilitate EMT and encourage osimertinib resistance. Furthermore, treatment with PIM1 inhibitors prevents EMT progression and re-sensitizes osimertinib-resistant NSCLC cells to osimertinib. PIM1/GSK3β signaling is activated in clinical samples of osimertinib-resistant NSCLC, and dual epidermal growth factor receptor (EGFR)/PIM1 blockade synergistically reverse osimertinib-resistant NSCLC in vivo. These data identify PIM1 as a driver of EMT-associated osimertinib-resistant NSCLC cells and predict that PIM1 inhibitors and osimertinib combination therapy will provide clinical benefit in patients with EGFR-mutant NSCLC.

## Introduction

Approximately 10–15% of Caucasian patients and up to 50% of East-Asian patients with non-small cell lung cancer (NSCLC) harbor epidermal growth factor receptor (EGFR) activating mutations [1, 2]. Osimertinib, a third-generation irreversible EGFR tyrosine kinase inhibitor (TKI), targets both EGFR-sensitive mutations (such as exon 19 deletions and L858R mutations) and EGFR T790M mutations, as the preferred treatment choice for EGFR-mutant NSCLC [3, 4]. However, resistance to osimertinib is inevitable, and further understanding of osimertinib resistance mechanisms is necessary. Osimertinib resistance can be broadly grouped into EGFR-dependent mechanisms, such as C797X mutations, and EGFR-independent mechanisms, such as the amplification of MET and HER2, epithelial-mesenchymal transition (EMT) and small cell lung cancer transformation [5–7]. The vast majority of resistance to osimertinib occurs via EGFR-independent mechanisms for which there are few targeted treatment options, such as EMT [8]. A thorough understanding of the signaling pathways that incorrectly regulate EMT is essential to overcoming osimertinib resistance.

EMT is a biological process characterized by the loss of polarity and cell-to-cell adhesion in epithelial cells, resulting in increased motility and invasion [9]. Routine testing for changes in gene or protein expression that indicate EMT is not commonly conducted, which may result in an underestimation of the incidence of this resistance mechanism. EMT is connected with resistance to multiple anti-cancer methods with different mechanisms of action, such as chemotherapy and targeted therapy [10, 11]. Up to 20% of patients resistant to EGFR TKIs, including third-generation inhibitors such as osimertinib, exhibit the EMT phenotype [12, 13]. Despite these revelations, in around two-thirds of patients remain unable it remains impossible to identify specific mechanisms of osimertinib resistance [14]. In the present study, we prove that signaling through proviral integration site for Moloney murine leukemia virus 1 (PIM1) can contribute to osimertinib resistance in a subset of NSCLC.

PIM1 is a serine/threonine kinase belonging to the calcium/calmodulin-regulated kinase (CAMK) family that is overexpressed or aberrantly expressed in NSCLC and plays a role in NSCLC proliferation, apoptosis, metastasis, and drug resistance [15–18]. Inhibiting PIM1 with shRNA or a PIM1 kinase inhibitor attenuates oncogenesis and EMT by downregulating downstream transcription factors [19, 20]. Glycogen synthase kinase 3β (GSK3β) is a direct substrate of PIM1, and its tumor-suppressive effects on cancer cells are blocked by PIM1-induced phosphorylation, leading to increased cell migration and adhesion [21]. It is unknown, however, whether PIM1 upregulation contributes to EMT-associated osimertinib resistance. Here, our research sheds more light on the molecular alterations of EMT-associated osimertinib resistance and reveals that signaling through PIM1 can contribute to osimertinib resistance in preclinical models. Dual inhibition of PIM1 and EGFR may be an effective clinical strategy for blocking or overcoming EMT-associated osimertinib resistance.

## Materials and methods

### Cell lines

The H1975 and PC9 cell lines were purchased from the Procell Life Science &Technology (Wuhan, China). The H1975 osimertinib resistant (H1975/OR) and PC9 osimertinib resistant (PC9/OR) cell lines were successfully established by subjecting parental H1975 and PC9 cells, respectively, to a gradually increasing concentration of osimertinib up to 1 μM for a duration exceeding 2 months. All cells were cultured in RPMI 1640 medium (Thermo Fisher Scientific, MA, USA) supplemented with 10% fetal bovine serum (FBS, Thermo Fisher Scientific, MA, USA). Incubation of all cell lines was carried out at 37 °C in a humidified atmosphere containing 5% CO_2_. All cell lines were verified as mycoplasma negative.

### Human samples

Three pairs of osimertinib pre-treatment and resistance tumor specimens were obtained from three patients diagnosed with advanced-stage EGFR-mutant NSCLC in Tianjin Medical University Cancer Institute and Hospital between 2017 and 2023. The CT examination images with diagnosis, tumor-adjacent tissues, and tumor tissues were analyzed in this study. This study was approved by the Research Ethics Committee of Tianjin Medical University Cancer Institute and Hospital. All samples were collected with informed consent from the patients and all examining procedures were performed with the approval of the internal review and ethics boards of the hospital (bc2021285).

### Reagents and antibodies

The 3-(4,5-dimethylthiazol-2-yl)-2,5-diphenyltetrazolium bromide (MTT), Cycloheximide (CHX), Carbobenzoxyl-L-leucyl-L-leucyl-L-leucine (MG132), GSK3β inhibitor (LiCl), osimertinib, and small-molecule PIM1 inhibitor (SGI-1776) were purchased from Selleckchem (Houston, TX, USA). Lipofectamine 2000 (Thermo Fisher Scientific, MA, USA) was utilized for transfection purposes. Supplementary Table S1 provides a detailed list of antibodies used.

### Plasmids

Full length human PIM1 cDNA (NM_002648.4) was subcloned into the pLVX-IRES-Neo vector (Genechem, Shanghai, China). Plasmids containing the wild-type PIM1 (PIM1 WT) or the kinase-dead mutant of PIM1 (PIM1 KD) were constructed following previously described methods [22, 23]. An empty vector was defined as a negative control (Vector). ShRNA targeting PIM1 was synthesized and inserted into the pLKO.1-puromycin lentiviral vector (Addgene) to generate the PIM1 knockdown vectors (shPIM1#1 and shPIM1#2). The scrambled shRNA was inserted into the pLKO.1-puromycin lentiviral vector and defined as a negative control (shControl). The shRNA sequences were shown in Supplementary Table S2. The plasmids containing the wild-type GSK3β (GSK3β WT) or the S9A mutant of GSK3β (GSK3β S9A) were obtained from VectorBuilder (Guangzhou, China). Small interfering RNAs (siRNAs) targeting GSK3β (siGSK3β) and negative control (siControl) were purchased from RIBOBIO (Guangzhou, China). The siRNA sequences were shown in Supplementary Table S3.

### Quantitative real‑time PCR (qRT‑PCR)

Total RNA was extracted using TRIzol reagent (Takara, Kyoto, Japan), followed by reverse transcription with PrimScript RT Master Mix (Takara, Kyoto, Japan). Subsequently, qRT-PCR was conducted using the SYBR Green PCR Kit (Takara, Kyoto, Japan). The cycling characteristics were as described below: 95 °C/5 s and 60 °C/34 s for 40 cycles. The mRNA level was normalized to GAPDH. The relative expression level was determined using 2^−ΔΔCt^. All reactions were performed in triplicate. The primer sequences for the target genes can be found in Supplementary Table S4.

### RNA sequencing and analysis

Total RNA was extracted from H1975/OR and H1975 cells. The RNA sequencing was conducted by GenePlus (Jiangsu, China). The gene expression data and clinical information for LUAD were downloaded from the TCGA database. The R software (version 4.0.2, R Foundation for Statistical Computing, Vienna, Austria). was used to conduct bioinformatics analyses. The DESeq2 R package was employed to identify differential expression genes (DEGs). Genes with an adjusted *p* < 0.05 and |log2 (fold change) | > 1 were considered differentially expressed. Volcano plot and heat map was performed using the ggplot2 R package. Gene set enrichment analysis (GSEA), Gene ontology (GO) and Kyoto Encyclopedia of Genes and Genomes pathway enrichment analysis were performed using the clusterProfiler R package.

### Colony formation assay

As for the colony formation assay, 1000 indicated cells were seeded in 6-well plates and cultivated for 14 days. After the incubation period, the cells were washed, fixed, and stained with 0.5% crystal violet (Solarbio, Beijing, China). This staining allows the visualization of cell colonies formed during the culture period.

### MTT assay

Cellular sensitivity to osimertinib and cell viability assay was assessed by MTT assay. Cells were planted into 96-well plates (2 × 10^3^ cells/well) and then incubated with different concentrations of osimertinib for 48 h. For the cell viability assay, cells were planted into 96-well plates (2 × 10^3^ cells/well) and then incubated for 0, 24, 48, 72, and 96 h. After the incubation period, 20 μl of MTT reagent applied to each well and incubated for 4 h. Then 150 μl of dimethyl sulfoxide (DMSO, Solarbio, Beijing, China) was added to dissolve the formazan crystals formed by viable cells. The absorbance was then measured at 490 nm using Gen5 data analysis software. The half maximal inhibitory concentration (IC_50_) was calculated using appropriate statistical methods. Graphs were plotted to visualize the results.

### Transwell assay

The transwell assay for cell migration or invasion was conducted using a 24-well culture insert. 3 × 10^4^ cells were suspended in 200 μl of serum-free RPMI-1640 media. The cell suspension was added to the upper chamber of the transwell insert, which was coated with or without a matrix component like Matrigel (BD Biosciences, NJ, USA) for invasion or migration assays. 500 μl of RPMI-1640 supplemented with 20% FBS was added to the lower chamber of the insert as a chemoattractant. The transwell chambers were incubated for 24 h to allow cells to migrate or invade through the porous membrane. After the incubation period, cells that did not migrate or invade through the membrane and remained on the upper surface of the chamber were carefully cleaned by gently wiping the surface with a cotton swab. The cells that passed through the membrane and reached the lower surface of the chamber were fixed in 4% paraformaldehyde (Solarbio, Beijing, China) and stained with crystal violet.

### Wound healing assay

The associated cells were seeded in a 6-well plate and cultured until nearly 100% confluency. Three parallel wounds were made on the cell monolayer using a 20 μl sterile pipette tip. This creates a gap or “scratch” in the cell layer. After creating the wounds, cells were washed with phosphate-buffered saline (PBS, Solarbio, Beijing, China) to remove any cellular debris or detached cells. Cells were then cultured on fresh medium without FBS. Images of each gap or wound were captured at the beginning of the experiment (0 h) and at the end of the experiment (24 h).

### Immunofluorescence

The related cells were initially seeded on cover glass placed in a 12-well plate and incubated overnight at 37 °C to stabilize and adhere to the surface. The cells were then treated with SGI-1776 or vehicle (DMSO) for 24 h. The cells were permeabilized for 15 min using 0.5% Triton X-100 (Solarbio, Beijing, China). Following permeabilization, the cells were fixed for 15 min using 4% paraformaldehyde. To prevent nonspecific binding, the cells were blocked for 1 h with 5% bovine serum albumin (BSA, Solarbio, Beijing, China). After blocking, the cells were treated with primary antibodies overnight at 4 °C. The next day, after removing the primary antibodies, the cells were incubated with secondary antibodies conjugated to fluorescent dyes like Alexa Fluor 488 or Alexa Fluor 594 (ZSGB-BIO, Beijing, China). The nucleus was stained with DAPI (Solarbio, Beijing, China) for 10 min. Images of the stained cells were captured using a Zeiss LSM880 confocal microscope (Zeiss, Germany) at a magnification of 630×. ImageJ software was employed to perform fluorescence colocalization analyses. The primary antibodies used were listed in Supplementary Table S1.

### Immunoblotting

Total protein was harvested using RIPA lysis buffer (Solarbio, Beijing, China). The protein concentration was determined with the Pierce BCA Protein Assay Kit (Thermo Fisher Scientific, MA, USA). The protein samples were separated on 10% SDS-PAGE gels and transferred to polyvinylidene fluoride (PVDF, Millipore, MA, USA) membranes. Following a blocking step with 5% skimmed milk powder for 1 h, the membranes were incubated with primary antibodies at 4 °C overnight. Afterward, the membranes were treated with secondary antibodies, either anti-rabbit or anti-mouse (ZSGB-BIO, Beijing, China), for 1 h at room temperature. Following a series of washes, specific proteins were detected with Western Lightning Plus-ECL (EpiZyme, Shanghai, China). The images were captured using a GelView 6000Plus system (Biolight Biotechnology, Guangzhou, China). The primary antibodies used were listed in Supplementary Table S1.

### Co-immunoprecipitation (co-IP) assay

For co-immunoprecipitation (co-IP) experiments, total protein was harvested in IP/Western lysing solution (Beyotime, Shanghai, China) containing a phosphatase inhibitor cocktail and a protease inhibitor cocktail. The lysates were then centrifuged at 12,000 × *g* for 15 min to remove cellular debris. The protein A/G magnetic beads (Thermo Fisher Scientific, MA, USA) were incubated with the respective antibodies on a rotating shaker at 4 °C for more than 6 h, followed by overnight incubation with protein extracts at 4 °C. On the following day, the immunoprecipitated protein complexes were collected after three washes with IP lysate and used for immunoblotting analysis. IgG was used as a negative control. Specific details on negative control IgG are provided in Supplementary Table S1.

### Ubiquitination assay

To investigate the endogenous ubiquitination of SNAIL and SLUG, H1975/OR and PC9/OR cells were transfected with either control shRNA (shControl) or PIM1 shRNA (shPIM1#1 and shPIM1#2) plasmids. The transfected cells were then treated with MG132 (10 μM) for 8 h. Subsequently, the cells were lysed using RIPA buffer. The proteins present in the cell lysate were then subjected to immunoprecipitation with anti-SNAIL and anti-SLUG antibodies to specifically isolate ubiquitinated forms of SNAIL and SLUG. The presence of endogenous ubiquitin chains in SNAIL and SLUG was assessed by immunoblotting analysis with an antibody targeting ubiquitin. The relevant antibodies used were listed in Supplementary Table S1.

### Animal experiments

To evaluate the impact of targeting PIM1 on osimertinib-resistant NSCLC, we implanted 4 × 10^6^ PC9/OR cells transfected with either control shRNA (shControl) or PIM1 shRNA (shPIM1) plasmids into the subcutaneous tissue of 6-week-old female nude mice (GemPharmatech, Jiangsu, China) to generate xenograft tumors. Each group consisted of 5 mice (*n* = 5). Once the tumor size reached approximately 60 mm^3^, the mice were administered either vehicle control (saline) or osimertinib (5 mg/kg body weight, once per day, oral gavage). During the experiment, the length (L) and width (W) of the tumors were measured using a caliper at predefined time points. The tumor volume was calculated using the formula: (L × W^2^)/2. At the conclusion of the study, the tumors were collected and weighed. They were then dissected, fixed, and embedded in paraffin for further analysis.

To investigate the impact of PIM1 inhibitors on osimertinib-resistant NSCLC, we implanted 4 × 10^6^ PC9/OR cells into the subcutaneous tissue of 6-week-old female nude mice to generate xenograft tumors. Each group consisted of 6 mice (*n* = 6). Once the tumor size reached approximately 60 mm^3^, the mice were randomly divided into 4 groups. They were administered vehicle control (saline), SGI-1776 (5 mg/kg body weight, once every 2 days, intraperitoneal injection), osimertinib (5 mg/kg body weight, once per day, oral gavage), or SGI-1776 combined with osimertinib. Tumor formation was monitored at specific time intervals during the experiment.

The metastatic ability of tumor cells was evaluated using a tail vein metastasis model in nude mice. To construct the lung metastasis model, 100 μl cell suspension containing 2 × 10^6^ PC9/OR cells was injected intravenously through tail vein and treatment was started on day 21 ending on day 40. Tumor metastasis was observed in vivo using an in vivo imaging system. Mice were imaged by intraperitoneal injection of 15 mg/mL D-Luciferin (APExBIO, Houston, USA). In vivo imaging was performed 10–15 min after the injection.

At the conclusion of the study, the xenograft tumor and lung of each mouse were harvested and analyzed. Subsequently, they underwent immunohistochemistry analysis for further investigation.

All animal experiments were carried out following the Guide for the Care and Use of Laboratory Animals and authorized by the Animal Ethical and Welfare Committee of Tianjin Medical University Cancer Institute and Hospital (AE-2022026).

### Hematoxylin-eosin stain (H&E stain) and immunohistochemistry (IHC)

The mouse xenograft tumor tissues were fixed in formalin, embedded in paraffin, and then sliced into sections of 4 µm. For H&E staining, deparaffinized slides were baked and passed through graded alcohol. They were stained with Harris hematoxylin for 5 min and 0.5% eosin (Solarbio, Beijing, China) for 1 min. For IHC staining, histopathological sections were baked overnight at 60 °C, followed by deparaffinization and dehydration. Antigen retrieval and blocking of endogenous peroxidase activity were performed. The slices were then incubated with various primary antibodies in a humidified container at 4 °C for 24 h. Afterward, they were incubated with corresponding secondary antibodies at room temperature for 1 h. Diaminobenzidine (DAB, ZSGB-BIO, Beijing, China) was then added for a 2-min incubation until a brown reaction was observed. Digital images were captured using a light microscope. The primary antibodies used were listed in Supplementary Table S1.

The results of the IHC assays were analyzed by two professional pathologists who were blinded to the experimental procedures. The frequency of positive cells was classified into five categories: 0 (negative), 1 (1–25%), 2 (26–50%), 3 (51–75%), and 4 (76–100%). The staining degree was categorized as follows: 0 (negative), 1 (weak), 2 (moderate), and 3 (strong). The IHC score was estimated by multiplying the two outcomes mentioned above.

### Statistical analysis

All assays were repeated at least three times. Two-tailed unpaired or paired Student’s *t* tests performed with GraphPad Prism software (Version 8.3, La Jolla, USA) were used for two group comparisons, while one-way or two-way ANOVA was used to compare multiple groups. *p* < 0.05 was viewed as significant (*p < 0.05, **p < 0.01, ***p < 0.001 and **** *p* < 0.0001), ns, no significance.

## Results

### Osimertinib-resistant NSCLC cells exhibit mesenchymal properties and increased expression of PIM1

**Fig. 1:**
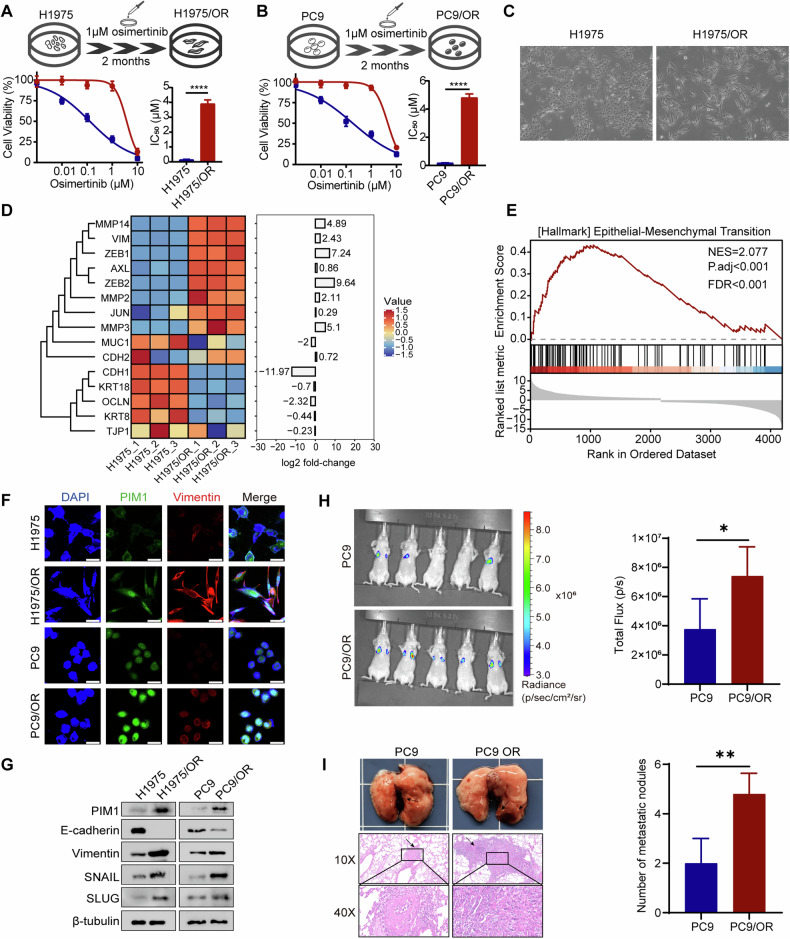
Upregulation of PIM1 expression and development of EMT are observed in osimertinib-resistant cells. **A**, **B** The IC_50_ values of parental (H1975 and PC9) and osimertinib-resistant (H1975/OR and PC9/OR) cells treated with osimertinib. Data are presented as mean ± SD, ****p < 0.0001, Student’s *t* test. **C** Morphology of H1975 and H1975/OR cells. **D** Heatmaps of DEGs related to mesenchymal markers and epithelial markers. **E** EMT pathway enrichment in H1975 cell lines as determined by GSEA. **F** Immunofluorescent staining of PIM1 and Vimentin in H1975/OR and PC9/OR cells compared with parental cells. Scale bar, 50 μm. **G** Detection of expression levels of EMT-associated molecules before and after osimertinib resistance by immunoblotting. **H** The fluorescence intensity of lung metastases was measured. Data are presented as mean ± SD, *p < 0.05, Student’s *t* test. **I** Macroscopic appearances of lung images of each group, black arrows indicate the tumor nodules. Lung metastasis nodules were confirmed by H&E staining. The number of lung metastatic nodules was measured. Data are presented as mean ± SD, **p < 0.01, Student’s *t* test.

Cell sensitivity to osimertinib was evaluated by the MTT assay, revealing significantly lower half-maximal inhibitory concentration (IC_50_) values for H1975 and PC9 cells compared to H1975/OR and PC9/OR cells (Fig. 1A, B). H1975/OR and PC9/OR cells were resistant to osimertinib. During cell culture stimulation with exposure to osimertinib, we noticed that the H1975/OR cells exhibited acquired prominent EMT features, such as cell long fusiform, cell detachment, and other distinctive traits (Fig. 1C). Several previous studies demonstrated a strong link between increased PIM1 expression and drug resistance, invasion, and metastasis in lung cancer [15–18]. The co-expression heatmap using TCGA-LUAD dataset uncovered a robust correlation between PIM1 expression and various EMT-associated molecules, including SNAIL, SLUG, Vimentin, and others (Fig. S1A). As a result, we performed transcriptome sequencing analysis on both H1975 and H1975/OR cells. We discovered 4586 DEGs, 2200 genes had a substantial down-regulation and 2386 genes had a significant up-regulation (Fig. S1B). Mesenchymal marker expression was much higher and epithelial marker expression was significantly lower in osimertinib-resistant cells (Fig. 1D). The analysis of differential gene pathway enrichment revealed a notable activation in the EMT pathway when osimertinib resistance emerged (Fig. 1E, Fig. S1C, Supplementary Tables S5 and S6). The immunofluorescence assay showed that PIM1 and Vimentin were mostly found together in the cytoplasm. This colocalization was particularly increased following resistance to osimertinib (Fig. 1F). Inspired by the co-expression heat map and GSEA results, we evaluated the protein levels of PIM1 and EMT-associated molecules protein levels in osimertinib-resistant cells. PIM1 protein level was significantly higher in osimertinib-resistant H1975/OR and PC9/OR cells than parental H1975 and PC9 cells. These resistant cells demonstrated a significant reduction in the expression of the epithelial marker E-cadherin, while showing a significant increase in the expression of the mesenchymal marker Vimentin, and transcription factors SLUG and SNAIL (Fig. 1G). Transwell assay indicated that osimertinib-resistant cells showed better migration and invasion ability (Fig. S1D). To verify the metastatic ability of osimertinib-resistant cells in vivo, luciferase-labeled PC9 and PC9/OR cells were injected into the tail vein of nude mice, and cell metastasis was monitored using bioluminescence imaging. The number of metastatic lung nodules was quantified after 6 weeks. Compared to the PC9 group, the PC9/OR group exhibited enhanced metastatic ability in vivo (Fig. 1H, I). Taken together, these observations demonstrate that the expression of PIM1 in osimertinib-resistant cells is associated with the acquisition of a mesenchymal phenotype.

### Inhibition of PIM1 reduces cell proliferation and migration in osimertinib-resistant cells, reversing drug resistance

**Fig. 2:**
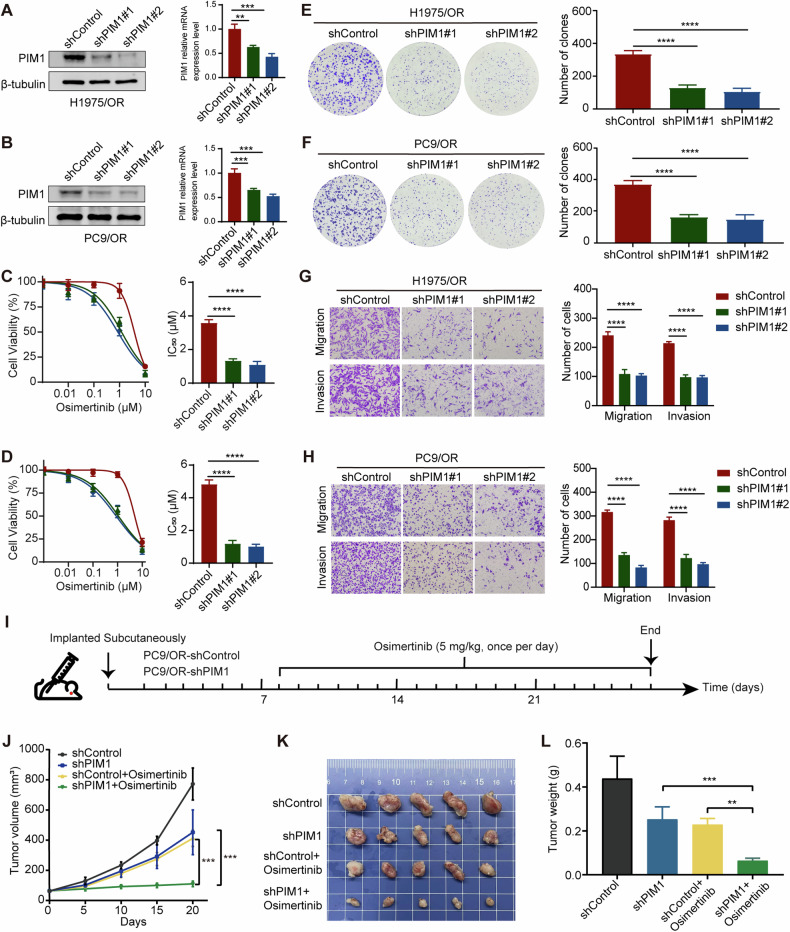
PIM1 inhibition is sufficient to overcome osimertinib resistance. **A**, **B** Stable PIM1 knockdown using shRNAs in H1975/OR and PC9/OR cells. Data are presented as mean ± SD, **p < 0.01, ***p < 0.001, One-way ANOVA. **C**, **D** Effect of PIM1 knockdown on IC_50_ values of osimertinib in H1975/OR and PC9/OR cells. Data are presented as mean ± SD, ****p < 0.0001, One-way ANOVA. **E**, **F** H1975/OR and PC9/OR colony formation abilities following PIM1 knockdown. Data are presented as mean ± SD, ****p < 0.0001, One-way ANOVA. **G**, **H** Influence of PIM1 knockdown on the migration and invasion abilities of H1975/OR and PC9/OR cells. Data are presented as mean ± SD, ****p < 0.0001, Two-way ANOVA. **I** Diagram of a mouse xenograft treated with osimertinib. **J** Tumor volume was measured on the indicated days. Data are presented as mean ± SD, ***p < 0.001, One-way ANOVA. **K** The tumors were dissected at the end of the experiment. **L** Measurement of tumor weights. Data are presented as mean ± SD, **p < 0.01, ***p < 0.001, One-way ANOVA.

To investigate the requirement of PIM1 in osimertinib resistance, we assessed whether genetic inhibition of PIM1 could restore sensitivity to osimertinib in H1975/OR and PC9/OR cells. Our experiments provided evidence that genetic inhibition of PIM1 in osimertinib-resistant cell lines indeed restored sensitivity to osimertinib (Fig. 2A–D). Cells with PIM1 knockdown demonstrated a significant decrease in clonal formation and cell viability compared to the control group when exposed to osimertinib (Fig. 2E, F, Fig. S2A, B). Transwell and wound healing assays revealed that PIM1 knockdown significantly reduced the migration and invasion abilities of H1975/OR and PC9/OR cells (Fig. 2G, H, Fig. S2C, D). After conducting the necessary in vitro studies, we investigated whether the notable effects of PIM1 knockdown on tumor response to osimertinib could be successfully replicated in vivo. For this purpose, PC9/OR cells were transfected with control shRNA (shControl) or shPIM1 and then injected subcutaneously into nude mice to establish tumor xenografts. Subsequently, the mice were administered either saline or osimertinib (5 mg/kg body weight, once per day, oral gavage) (Fig. 2I). In line with our in vitro discoveries, we observed that despite the resistance of PC9/OR tumors to osimertinib, the suppression of PIM1 considerably intensified the inhibitory of osimertinib in vivo. This was evidenced by a notable reduction in tumor size and weight (Fig. 2J–L). No significant difference in mouse body weight was observed between control and osimertinib-treated mice under the tested conditions (Fig. S2E). Furthermore, an immunohistochemistry assay revealed a pronounced decrease in Vimentin staining intensity in the shPIM1 group (Fig. S2F). These findings demonstrate that PIM1 plays a critical role in mediating osimertinib resistance and its genetic inhibition may provide a potential therapeutic strategy to overcome this resistance. Next, we wanted to investigate the underlying mechanisms of PIM1-mediated osimertinib resistance in NSCLC patients.

### PIM1 suppresses the ubiquitination-mediated proteasomal degradation of SNAIL and SLUG

**Fig. 3:**
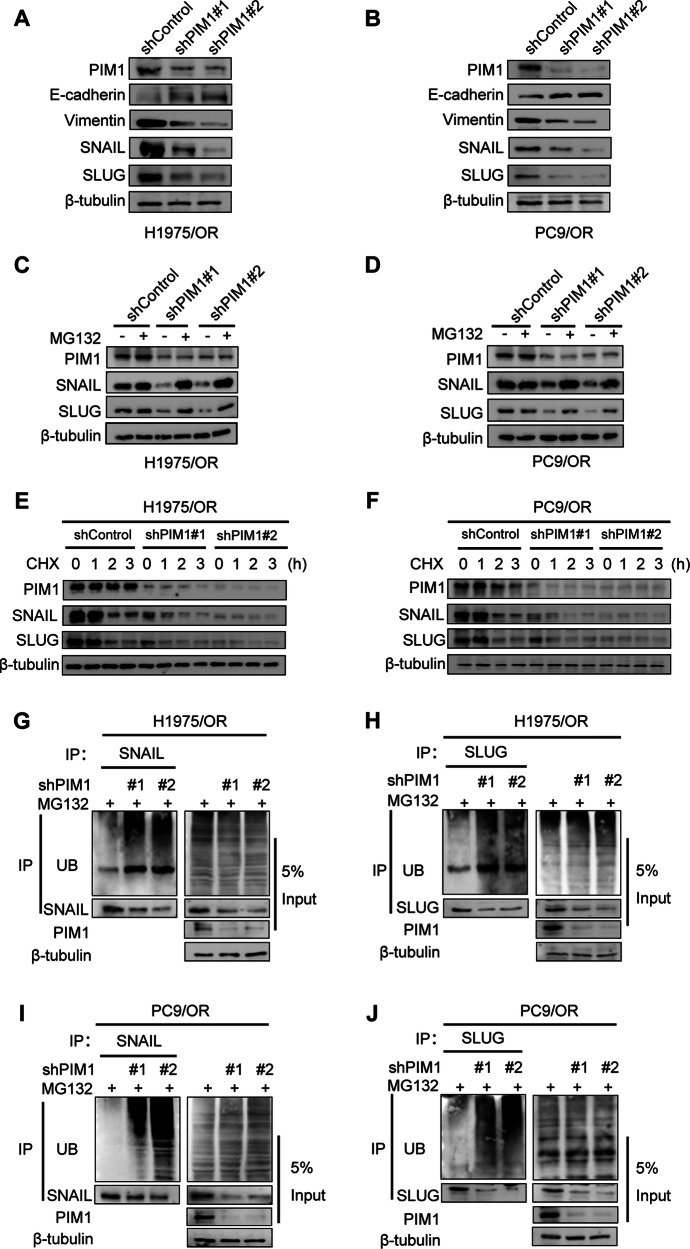
PIM1 promotes SNAIL and SLUG stabilization and deubiquitination. **A**, **B** The impact of PIM1 knockdown on EMT-associated molecules expression was assessed by immunoblotting in H1975/OR and PC9/OR cells. **C**, **D** Immunoblotting was used to determine the impact of PIM1 knockdown and the addition of MG132 (10 μM, 24 hours) on SNAIL and SLUG expression in H1975/OR and PC9/OR cells. **E**, **F** Assessment of protein half-life of SNAIL and SLUG in PIM1 knockdown H1975/OR and PC9/OR cells treated with CHX (20 μg/mL) for 0, 1, 2, and 3 h using a CHX pulse-chase assay. **G**-**J** The ubiquitination assay was performed to assess the influence of PIM1 knockdown on the levels of ubiquitination of SNAIL and SLUG in H1975/OR and PC9/OR cells.

To investigate the regulatory role of PIM1 on EMT in osimertinib-resistant cells, we stably knocked down PIM1 in H1975/OR and PC9/OR cell lines. The previously upregulated mesenchymal markers and transcription factors, such as Vimentin, SLUG, and SNAIL, showed a downregulation upon PIM1 knockdown, while E-cadherin expression was restored (Fig. 3A, B). These findings were consistent with previous research, indicating that inhibiting PIM1 impeded EMT progression [20]. It is pertinent to highlight that the knockdown of PIM1 resulted in a specific reduction in the levels of SNAIL and SLUG proteins, without any notable alterations in mRNA expression levels (Fig. S3A, B). This suggested that PIM1 might regulate SNAIL and SLUG expression through a post-translational mechanism. Interestingly, the repression of SNAIL and SLUG caused by PIM1 suppression was counteracted by the introduction of the proteasome inhibitor MG132. This indicated that PIM1 hindered the degradation of SNAIL and SLUG mediated by the proteasome (Fig. 3C, D). Our second step was to block protein synthesis by using CHX pulse-chase assay to determine whether PIM1 regulates SNAIL and SLUG stability, demonstrating that knockdown of PIM1 decreased the half-life of endogenous SNAIL and SLUG. The destabilization of SNAIL and SLUG proteins led to an accelerated degradation process (Fig. 3E, F). To expand on these findings, we conducted endogenous ubiquitination experiments, which showed that knockdown of PIM1 led to increased ubiquitination of endogenous SNAIL and SLUG proteins (Fig. 3G–J). These data suggest that PIM1 inhibits SNAIL and SLUG ubiquitination degradation.

### PIM1 induces GSK3β phosphorylation in osimertinib-resistant NSCLC

**Fig. 4:**
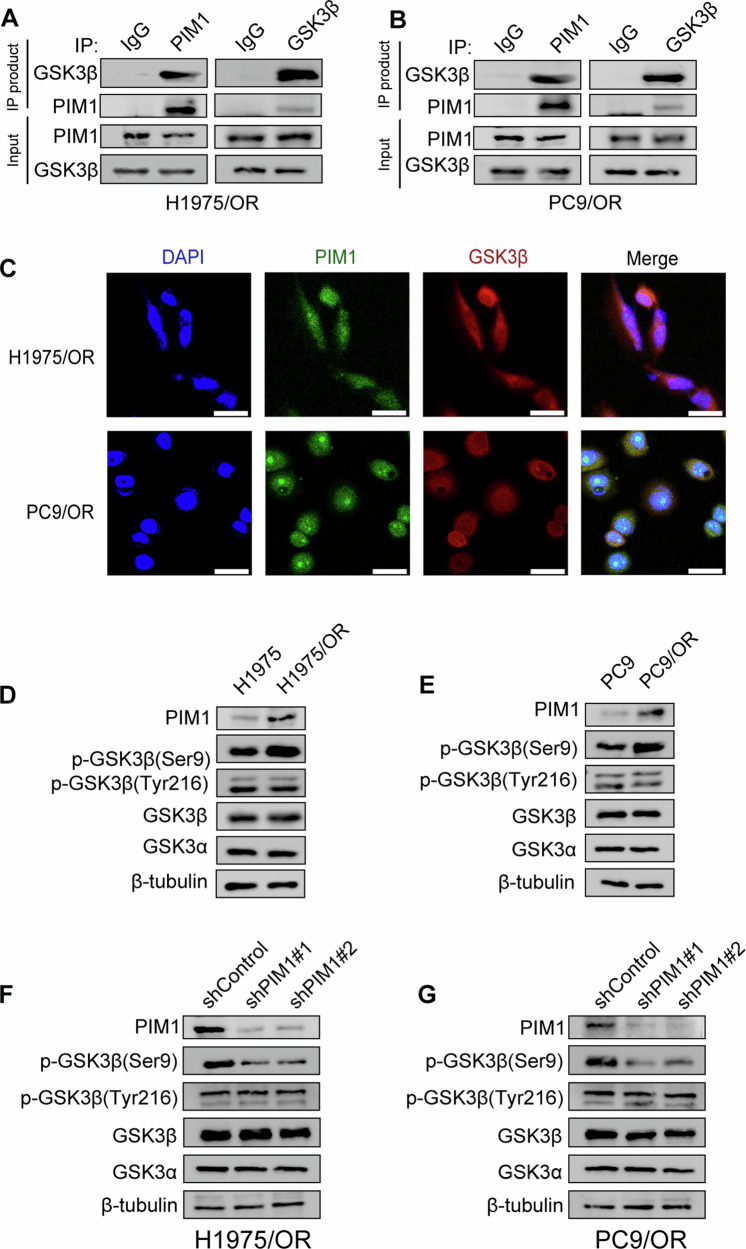
Phosphorylation of GSK3β Ser9 site by PIM1 kinase inhibits GSK3β activity. **A**, **B** Demonstration of PIM1 and GSK3β interaction in H1975/OR and PC9/OR cells through a co-IP assay. **C** Immunofluorescent co-localization analysis of PIM1 and GSK3β in H1975/OR and PC9/OR cells. Scale bar, 50 μm. **D**, **E** Detection of p-GSK3β (Ser9), GSK3β, GSK3α, and p-GSK3β (Tyr216) expression levels before and after osimertinib resistance via immunoblotting. **F**, **G** Efficacy of PIM1 knockdown on the expression levels of p-GSK3β (Ser9), GSK3β, GSK3α, and p-GSK3β (Tyr216) analyzed by immunoblotting in H1975/OR and PC9/OR cells.

Phosphorylation of GSK3β Ser9 site by PIM1 kinase inhibited GSK3β activity [21]. The research confirmed the endogenous interaction between PIM1 and GSK3β through co-IP experiments performed in H1975/OR and PC9/OR cells (Fig. 4A, B). Moreover, immunofluorescence staining indicated that PIM1 and GSK3β co-localized in both the cytoplasm and nucleus of H1975/OR and PC9/OR cells (Fig. 4C). The expression levels of p-GSK3β (Ser9), which was an inactive form of GSK3β, were found to be significantly higher in H1975/OR and PC9/OR cells compared to parental cells. However, there were no significant variations observed in the expression levels of p-GSK3β (Tyr216), which was the active form of GSK3β, as well as GSK3β and GSK3α (Fig. 4D, E). After PIM1 knockdown, p-GSK3β (Tyr216), GSK3β, and GSK3α expression levels remained unchanged. But, notably, p-GSK3β (Ser9) expression levels were significantly reduced (Fig. 4F, G). These findings contribute to our understanding of the relationship and interplay between PIM1 and GSK3β in osimertinib-resistant NSCLC cells.

### Phosphorylation of the GSK3β Ser9 site by PIM1 contributes to the maintenance of SNAIL and SLUG stability

**Fig. 5:**
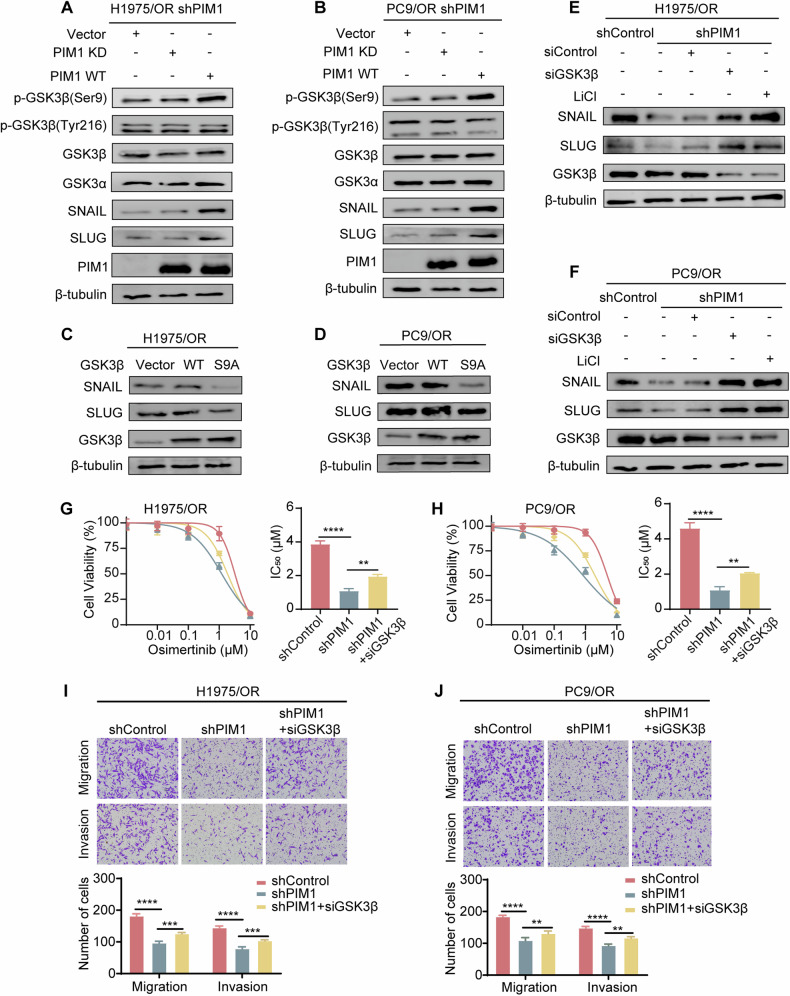
Phosphorylation of the GSK3β Ser9 site by PIM1 suppresses SNAIL and SLUG degradation. **A**, **B** The influence of PIM1 knockdown and restoration on p-GSK3β (Ser9), p-GSK3β (Tyr216), GSK3β, GSK3α, SNAIL and SLUG was analyzed by immunoblotting. **C**, **D** Analysis of SNAIL and SLUG expression via immunoblotting in H1975/OR and PC9/OR cells transfected with GSK3β WT and GSK3β S9A plasmids. **E**, **F** The efficacy of PIM1 knockdown and the addition of siGSK3β or LiCl on the expression levels of SNAIL and SLUG in H1975/OR and PC9/OR cells was determined by immunoblotting. **G**, **H** IC_50_ assay of H1975/OR-shPIM1 cells and PC9/OR-shPIM1 transfected with siGSK3β. Data are presented as mean ± SD, **p < 0.01, ****p < 0.0001, One-way ANOVA. **I**, **J** Transwell assay of H1975/OR-shPIM1 cells and PC9/OR-shPIM1 transfected with siGSK3β. Data are presented as mean ± SD, **p < 0.01, ***p < 0.001, **** *p* < 0.0001, Two-way ANOVA.

In order to further examine the impact of PIM1-mediated phosphorylation on GSK3β activity, we designed and constructed plasmids of PIM1 WT and PIM1 KD. The expression levels of p-GSK3β (Ser9), SNAIL, and SLUG were diminished when PIM1 was knocked down. However, this decrease in expression could be reversed by transfection with PIM1 WT, but not by transfection with PIM1 KD. The kinase-death mutations of PIM1 contribute to its loss of regulatory function in GSK3β phosphorylation (Fig. 5A, B). GSK3β is a crucial protein that interacts with SNAIL and SLUG, leading to phosphorylation-dependent proteasome degradation of both SNAIL and SLUG [24–26]. To gain insight into the function of GSK3β in regulating EMT and the mechanism behind osimertinib resistance, we created two types of plasmids: GSK3β WT and GSK3β S9A (site-directed mutagenesis to change Ser9 to an alanine residue that cannot be phosphorylated). PIM1 was responsible for phosphorylating the Ser9 site of GSK3β, leading to the inactivation of its function. When GSK3β was phosphorylated, it became inactive form and lost its ability to degrade SNAIL and SLUG through ubiquitination. When the GSK3β WT plasmid was introduced, PIM1 continued to phosphorylate the exogenous addition of GSK3β. As a result, the levels of SNAIL and SLUG expression in the GSK3β WT group were similar to those in the Vector group. However, GSK3β S9A remained unaffected by PIM1-mediated phosphorylation, which maintained its ability to degrade SNAIL and SLUG via ubiquitination. The levels of SNAIL and SLUG expression in the GSK3β S9A group were notably lower than in both the Vector and GSK3β WT groups (Fig. 5C, D). These data suggested that PIM1 maintained SNAIL and SLUG protein stability by phosphorylating GSK3β Ser9 sites. Inhibition of PIM1 significantly increased SNAIL and SLUG ubiquitination and degradation. To gain a deeper understanding of the mechanisms by which PIM1 regulates the EMT process, siGSK3β and LiCl were separately introduced into PIM1 knockdown H1975/OR and PC9/OR cells. It is worthwhile to mention that the introduction of siGSK3β and LiCl effectively reversed the decrease in SNAIL and SLUG expression caused by PIM1 knockdown (Fig. 5E, F). We then performed an IC_50_ assay. As compared to control cells, the results demonstrated that knockdown of PIM1 greatly improved osimertinib sensitivity in H1975/OR and PC9/OR cells. However, the impact of shPIM1-induced effects on IC_50_ was partially reversed when transfected with siRNA targeting GSK3β (Fig. 5G, H). Transfection of siGSK3β also restores migration and invasion ability of H1975/OR and PC9/OR after PIM1 knockdown (Fig. 5I, J). Collectively, these results indicate that the influence of ubiquitination on SNAIL and SLUG stability can be regulated by kinase-mediated phosphorylation, suggesting that PIM1 controls the protein level of SNAIL and SLUG in such a manner.

### Treatment with PIM1 inhibitor re-sensitizes osimertinib-resistant cells to EGFR TKIs

**Fig. 6:**
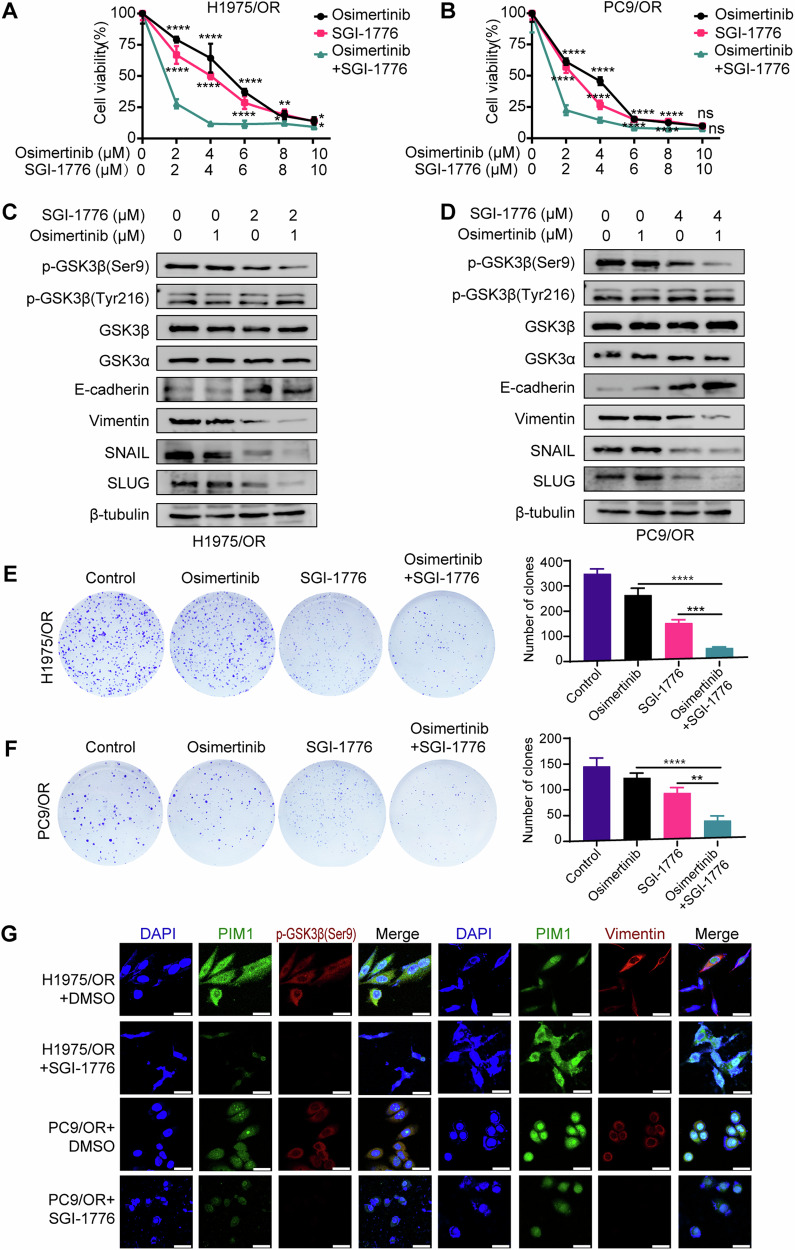
The combination of PIM1 inhibitor and osimertinib synergistically reverses osimertinib-resistant cells in vitro. **A**, **B** Cell viability measurement by MTT assay in H1975/OR and PC9/OR cells co-treated with osimertinib and SGI-1776 at the indicated concentrations for 48 h. Data are presented as mean ± SD, *p < 0.05, **p < 0.01, ****p < 0.0001, ns, no significance, One-way ANOVA. **C**, **D** Immunoblotting analysis of p-GSK3β (Ser9), p-GSK3β (Tyr216), GSK3β, GSK3α, and EMT-associated molecules expression in H1975/OR and PC9/OR cells co-treated with osimertinib and/or SGI-1776 at the indicated concentrations for 24 h. **E**, **F** Clonogenic assay of H1975/OR cells and PC9/OR cells co-treated with osimertinib and SGI-1776 at the indicated concentrations. Data are presented as mean ± SD, **p < 0.01, ***p < 0.001, ****p < 0.0001, One-way ANOVA. **G** Immunofluorescence analysis of PIM1, p-GSK3β (Ser9), and Vimentin in H1975/OR and PC9/OR cells after 24 h of SGI-1776 or DMSO treatment. Scale bar, 50 μm.

After studying the aforementioned findings, our objective was to explore the impact of pharmacologically inhibiting PIM1 on osimertinib-resistant cells. Treatment of osimertinib-resistant cells with the PIM1 specific inhibitor, SGI-1776, at doses that inhibit the PIM1 activity. We hypothesized that treatment with SGI-1776 would re-sensitize osimertinib-resistant cells to osimertinib treatment. Indeed, the group of subjects that received a combination of osimertinib and SGI-1776 exhibited a significant reduction in cell viability compared to the group that received only monotherapy (Fig. 6A, B). This suggested that the concurrent administration of osimertinib and SGI-1776 had a pronounced effect on suppressing osimertinib-resistant cells viability in a dose-dependent manner. Our study devised a concentration gradient for SGI-1776 ranging from 1 μM to 10 μM. The objective was to identify the most effective concentration for immunoblotting analysis. The findings of our research revealed that treating H1975/OR cells with 2 μM of SGI-1776, as well as administering 4 μM of SGI-1776 to PC9/OR cells, led to a partial decrease in the expression of p-GSK3β (Ser9) and EMT signaling pathway. Although treatment with SGI-1776 also reduced downstream signaling pathways, the combination of osimertinib and SGI-1776 was more effective in this regard than treatment alone (Fig. 6C, D). The combination treatment group was more effective in reducing colony counts than the SGI-1776 or osimertinib monotherapy group (Fig. 6E, F). Moreover, the immunofluorescence findings demonstrated a notable reduction in the co-localization of PIM1, p-GSK3β (Ser9), and Vimentin when SGI-1776 was introduced to H1975/OR and PC9/OR cells (Fig. 6G). Thus, SGI-1776 treatment re-sensitized osimertinib-resistant cells to osimertinib treatment, and the combination of SGI-1776 and osimertinib provided enhanced therapeutic benefit relative to monotherapies in osimertinib-resistant cell cultures.

### PIM1/GSK3β signaling is activated in clinical samples of osimertinib-resistant NSCLC

**Fig. 7:**
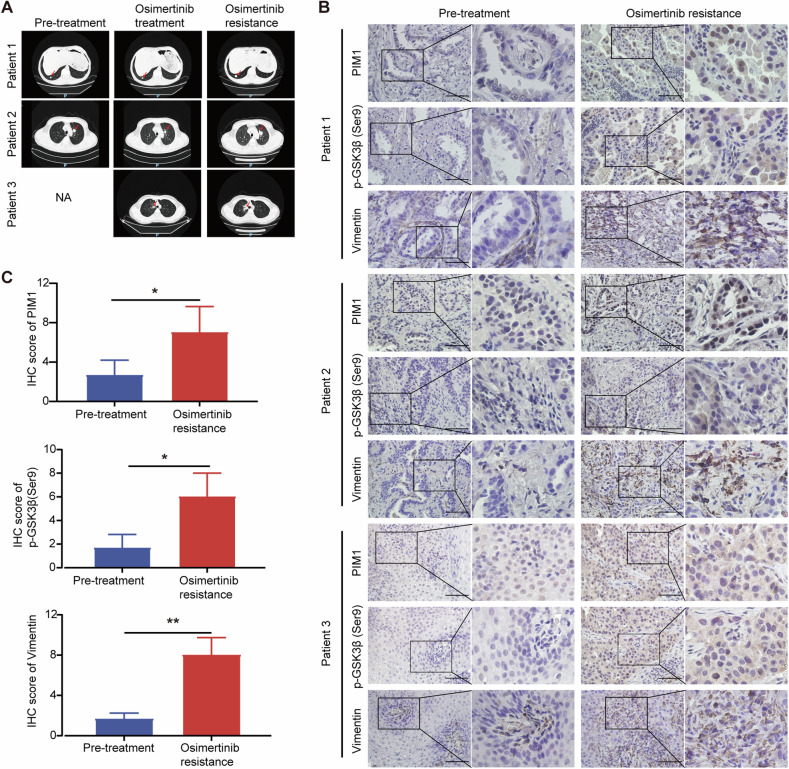
PIM1/GSK3β signaling is activated in clinical samples of osimertinib-resistant NSCLC. **A** CT images of lung tumor images before and after treatments. The red arrow indicated tumors. NA, not available. **B** PIM1, p-GSK3β (Ser9) and Vimentin staining in tumor specimens from the same patient before and after treatments. Scale bar, 25 µm. **C** PIM1, p-GSK3β (Ser9) and Vimentin IHC staining scores of patients before and after osimertinib resistance. Data are presented as mean ± SD, *p < 0.05, **p < 0.01, Student’s *t* test.

To establish the clinical relevance of increased PIM1/GSK3β signaling in mediating acquired resistance to osimertinib in NSCLC, we obtained CT images of three cases of advanced EGFR-mutant lung adenocarcinoma. Initially diagnosed with lung adenocarcinoma, these patients underwent genetic testing that revealed EGFR mutations (Fig. 7A, left panel). Treatment with the third-generation EGFR-TKI osimertinib resulted in partial remission (Fig. 7A, middle panel). However, these patients eventually developed acquired resistance over time as reflected by the progressive disease (Fig. 7A, right panel). Immunostaining of tumor tissue sections from lung biopsies showed weak positive signals for PIM1, p-GSK3β (Ser9), and Vimentin before treatment, which subsequently became strong positive signals upon development of acquired osimertinib resistance (Fig. 7B, C).

### Dual EGFR/PIM1 blocking reverses osimertinib resistance in vivo

**Fig. 8:**
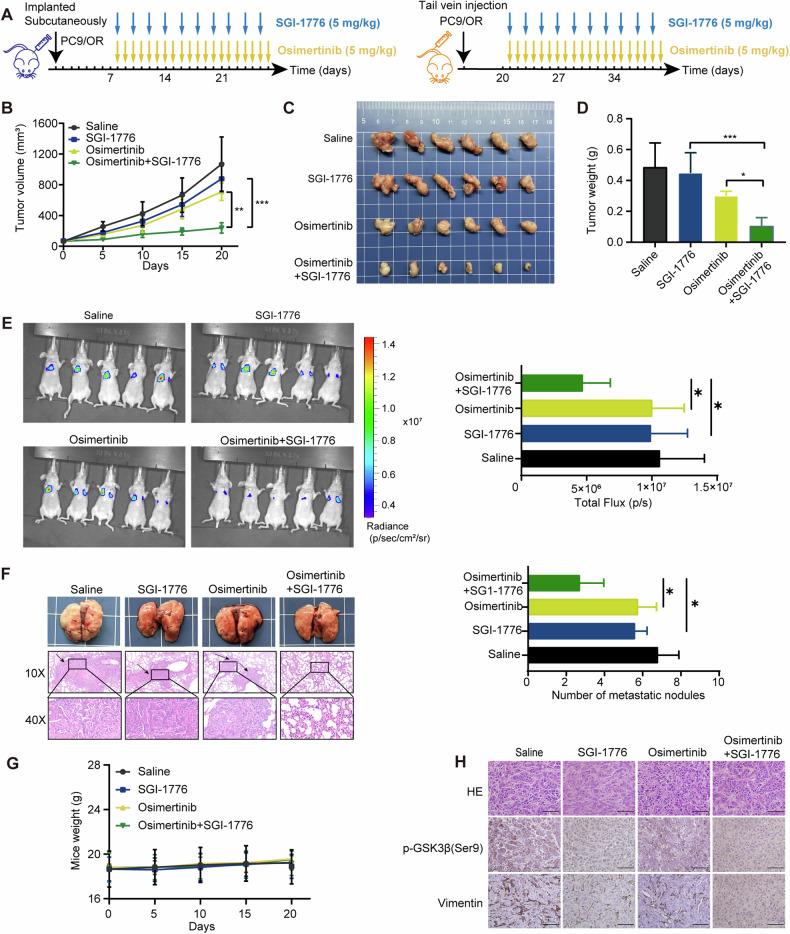
Dual EGFR/PIM1 blocking reverses osimertinib resistance in vivo. **A** Diagram of mouse model treated with osimertinib and SGI-1776. **B** Tumor volume was measured on the indicated days. Measurement of tumor weights. Data are presented as mean ± SD, **p < 0.01, ***p < 0.001, One-way ANOVA. **C** Tumors were dissected at the end of the experiment. **D** Measurement of tumor weights. Data are presented as mean ± SD, *p < 0.05, ***p < 0.001, One-way ANOVA. **E** The fluorescence intensity of lung metastases was measured. Data are presented as mean ± SD, *p < 0.05, One-way ANOVA. **F** Macroscopic appearances of lung images of each group, black arrows indicate the tumor nodules. Lung metastasis nodules were confirmed by H&E staining. The number of lung metastatic nodules was measured. Data are presented as mean ± SD, *p < 0.05, One-way ANOVA. **G** Measurement of mice weights. **H** H&E staining, p-GSK3β (Ser9) and Vimentin IHC staining of resected tumor tissues. Scale bar, 25 µm.

Tumor xenografts and lung metastasis model were established to assess the impact of SGI-1776 and osimertinib on osimertinib-resistant tumor growth and metastasis. The nude mice were randomly divided into four groups. Each group received a specific treatment after tumor development: osimertinib alone (5 mg/kg body weight, once per day, oral gavage), SGI-1776 alone (5 mg/kg body weight, once every 2 days, intraperitoneal injection), a combination of both drugs, or a control treatment (Fig. 8A). The administration of either osimertinib or SGI-1776 individually resulted in a marginal decrease in tumor growth and metastasis when compared to the control group. However, the combined application of osimertinib and SGI-1776 exhibited a notable and substantial inhibition in tumor growth and metastasis (Fig. 8B–F). The potential toxicity of the osimertinib and SGI-1776 combination therapy was evaluated by monitoring the body weights of the mice in each group. Throughout the treatment period, the body weights of the mice remained relatively stable, providing evidence that there was no significant toxicity associated with the combined treatment (Fig. 8G). Moreover, the analysis of immunohistochemistry demonstrated a notable reduction in the levels of Vimentin and p-GSK3β(Ser9) following the administration of osimertinib in combination with SGI-1776 (Fig. 8H). These findings provide additional support for the idea that inhibiting PIM1 could be a potential strategy to overcome osimertinib-resistant NSCLC.

## Discussion

Osimertinib has emerged as the preferred first-line treatment for EGFR-mutant NSCLC, owing to its superior effectiveness and enhanced overall survival compared to earlier generation EGFR TKIs [27]. The emergence of acquired resistance to EGFR inhibitors is almost universally observed in NSCLC patients with EGFR-activating mutations [5]. A more profound understanding of the molecular mechanisms that contribute to osimertinib resistance potentially provides strategies to enhance patient therapeutic response and survival. In the present investigation, we discover that PIM1 drives EMT-associated osimertinib resistance, thus suggesting that dual EGFR/PIM1 blockade is a promising clinical strategy for preventing and overcoming EMT-associated osimertinib resistance.

EMT has been observed in both pre-clinical and clinical resistance models [28]. The transformation of tumor cells from an epithelial to a mesenchymal phenotype enhances the invasiveness and metastatic ability of the tumor and leads to resistance to certain treatment agents, including EGFR-TKIs [29, 30]. This is consistent with our experimental results, where NSCLC cells gradually exhibited mesenchymal characteristics and significant activation of the EMT pathway during stimulation and culture with osimertinib. Our previous research revealed that PIM1 may serve as a viable therapeutic target for NSCLC [15, 31]. This study is the first to establish that PIM1 regulates cell viability and invasiveness in the context of EMT-associated EGFR TKI resistance. Importantly, we demonstrate that inhibition of PIM1 is sufficient to suppress the growth and metastasis of EGFR TKI-resistant cells and tumors, hinder EMT progression, and also restore sensitivity to osimertinib in multiple osimertinib-resistant cell lines.

GSK3β belongs to the serine/threonine kinase family and is involved in tumor development and drug resistance [32]. The phosphorylation of GSK3β Ser9 site holds substantial importance in governing its activity. The phosphorylation of GSK3β Ser9 site by PIM1 kinase inhibits GSK3β activity, thereby promoting the migration and invasion of prostate cancer cells [21]. Recent studies have implicated increased GSK3β phosphorylation as a mediator of acquired resistance to earlier generation EGFR TKIs [25, 33]. Studies conducted in the past have demonstrated a correlation between GSK3β Ser9 phosphorylation and SNAIL and SLUG expression in NSCLC [26, 34]. Beta-transducin repeats-containing proteins (β-TrCP) is known to be an E3 ubiquitin ligase. It has been reported to play a role in many GSK3β-degraded substrates [35]. SNAIL and SLUG is phosphorylated by GSK3β and subsequently undergoes β-Trcp-dependent ubiquitination and proteosomal degradation, which regulate E-cadherin expression and EMT [36, 37]. Our research findings indicate that in osimertinib-resistant NSCLC, there is an upregulation of PIM1 expression, which is sufficient to induce the inactivation (phosphorylation) of GSK3β. As a consequence of this inactivation, the ubiquitination degradation of SNAIL and SLUG is suppressed, leading to the stabilization and accumulation of SNAIL and SLUG proteins, ultimately resulting in EMT.

Abnormal expression of PIM1 kinase in various cancers has prompted extensive research into small molecule inhibitors targeting PIM1 proteins, with several of these inhibitors advancing to clinical trials [38]. Currently, the majority of clinical trials are focused on hematological disorders. For instance, SGI-1776, LGH447, AZD1208 and SEL-24 have been utilized in clinical trials for acute myeloid leukemia [39, 40]. In the case of multiple myeloma, Uzansertib LGH447, and TP-3654 have been studied, while lymphoma trials have employed Uzansertib and AZD1208 [41, 42]. It is important to note, however, that these studies are currently in phase I/II, certain challenges still persist. Hematologic toxicity, particularly thrombocytopenia, and anemia, remains a significant cause of severe adverse events, with reported instances of fatalities at high doses (NCT01456689). Some trials have been prematurely terminated due to poor tolerability (NCT02144038). It is currently unclear whether this is a molecule-specific toxicity or a mechanism-based toxicity. Despite these challenges, development of other PIM1 inhibitors is ongoing. Regretfully, as of now, there are no ongoing clinical studies focusing on lung cancer. Several studies have also demonstrated that the pan-PIM kinase inhibitor SGI-1776 exhibits significant anti-cancer activity [43, 44]. Here, we identify that SGI-1776 exhibited effective inhibition of GSK3β phosphorylation and suppressed SNAIL and SLUG in osimertinib-resistant NSCLC cells. Significantly, both in vitro and in vivo, it is observed that the combination of SGI-1776 and osimertinib synergistically inhibited tumor growth and reversed resistance in osimertinib-resistant NSCLC cells. This suggests that PIM1 inhibitor may serve as an effective strategy for overcoming acquired osimertinib resistance. One limitation of the translational application of this study in a clinical setting is that the assessment of EMT and the PIM1/GSK3β pathway is not a routine practice in patients when resistance occurs. Consequently, it becomes difficult to determine the frequency at which the mechanism described in this study, using cell line models, contribute to resistance. Additionally, the tumor microenvironment can impact EMT and therapeutic resistance, and the use of in vitro or xenograft models in immunodeficient mice may not accurately mimic the tumor microenvironment observed in patients who have developed acquired resistance to EGFR inhibitors.

**Fig. 9:**
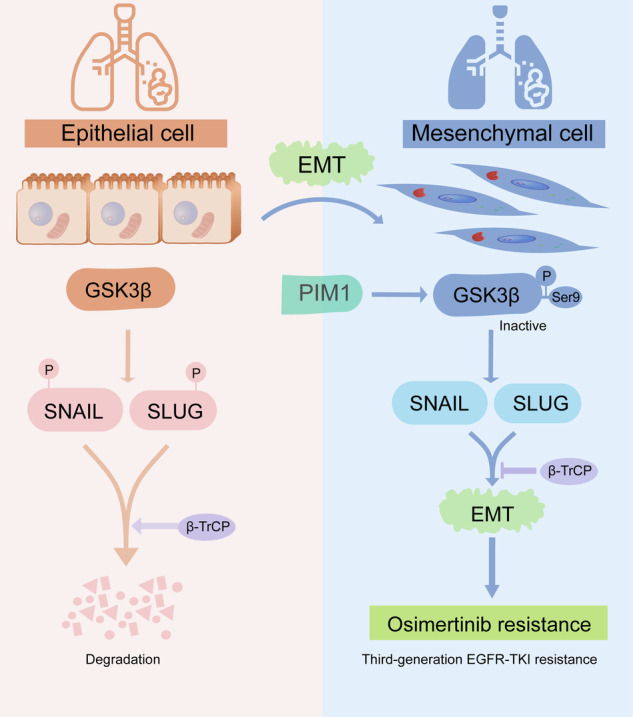
Mechanisms of the PIM1 kinase signaling pathway regulates EMT-associated osimertinib-resistant NSCLC. PIM1 suppresses the ubiquitin-proteasome degradation of SNAIL and SLUG by deactivating GSK3β through phosphorylation. The stability and accumulation of SNAIL and SLUG facilitate EMT and encourage osimertinib resistance.

In summary, this study reveals a novel mechanism by which PIM1 regulates EMT, and elucidates a critical role for the PIM1/GSK3β/SNAIL and SLUG axis in patients with EGFR-mutant NSCLC with acquired resistance to osimertinib (Fig. 9). Based on this remarkable finding, we identify PIM1 as a driver of EMT-associated osimertinib-resistant NSCLC cells and predict that dual EGFR/PIM1 blockade is an effective strategy to prevent and overcome EMT-associated osimertinib resistance.

## Supplementary information

Supplementary Figures

Supplementary Tables

Original western blots

## Data Availability

The data that support the findings of this study are available from the corresponding author upon reasonable request.
