# Supplementary material for: PIM1 kinase promotes EMT-associated osimertinib resistance via regulating GSK3β signaling pathway in EGFR-mutant non-small cell lung cancer
Source: Cell Death Dis. 2024 Sep 3;15(9):644. doi: 10.1038/s41419-024-07039-0 (PMC11372188; doi:10.1038/s41419-024-07039-0)
Supplement: Supplementary file 1 — Supplementary Figures [file 41419_2024_7039_MOESM1_ESM.docx]

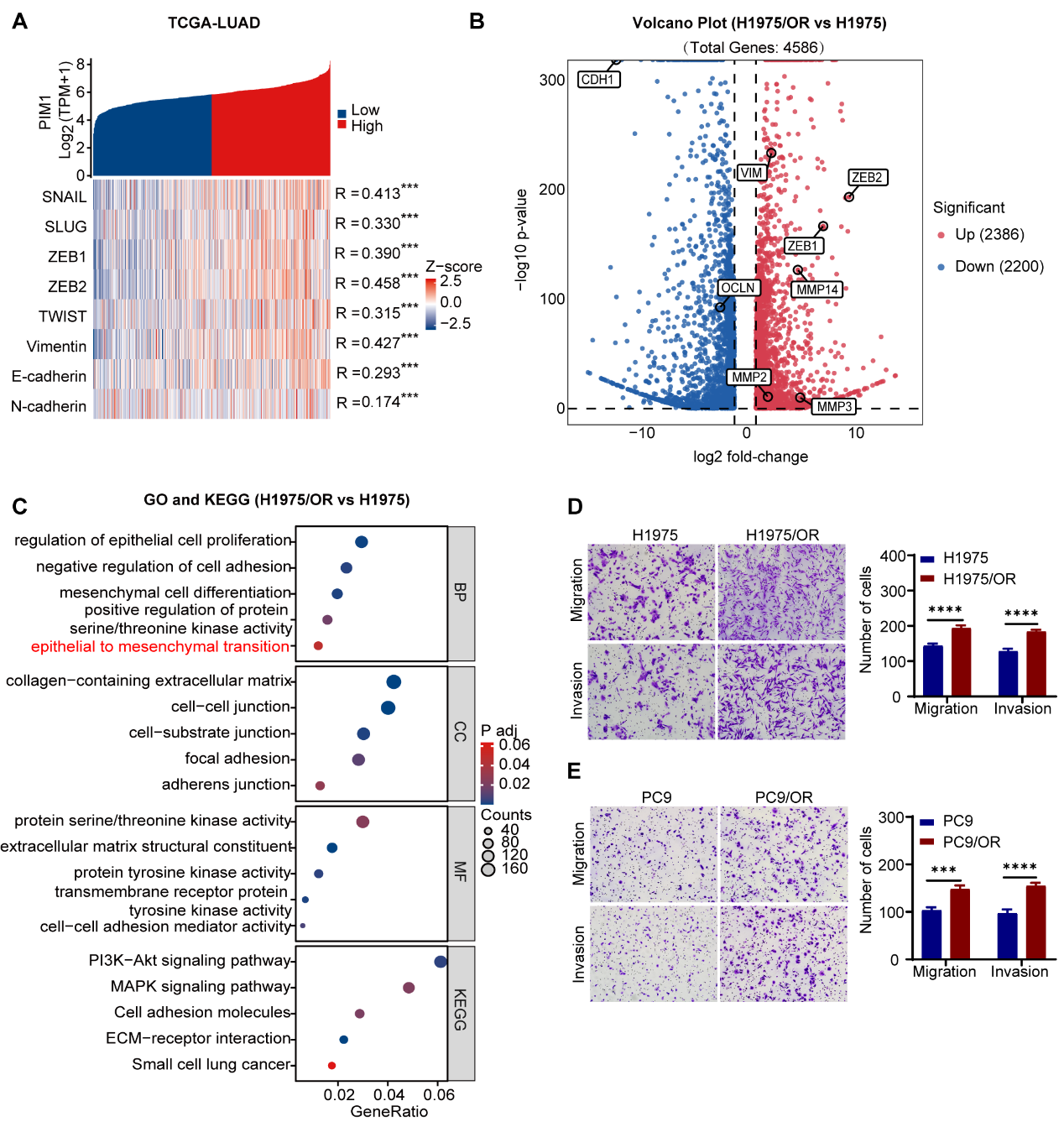


**Fig. S1.** (A) PIM1 was strongly correlated with EMT-associated molecules in lung adenocarcinoma samples. (B) Volcano plot showing the log2 fold-change and significance (−log10 p-value) of the transcriptome sequencing analysis. Red dots represent significantly upregulated genes, while the blue dots represent the significantly downregulated genes. (C) GO and KEGG enrichment analysis results in H1975 cell lines. (D, E) Comparison of migration and invasion ability between parental and osimertinib-resistant cells. Data are presented as mean ± SD, ****p* < 0.001, *****p*<0.0001, Two-way ANOVA.

**
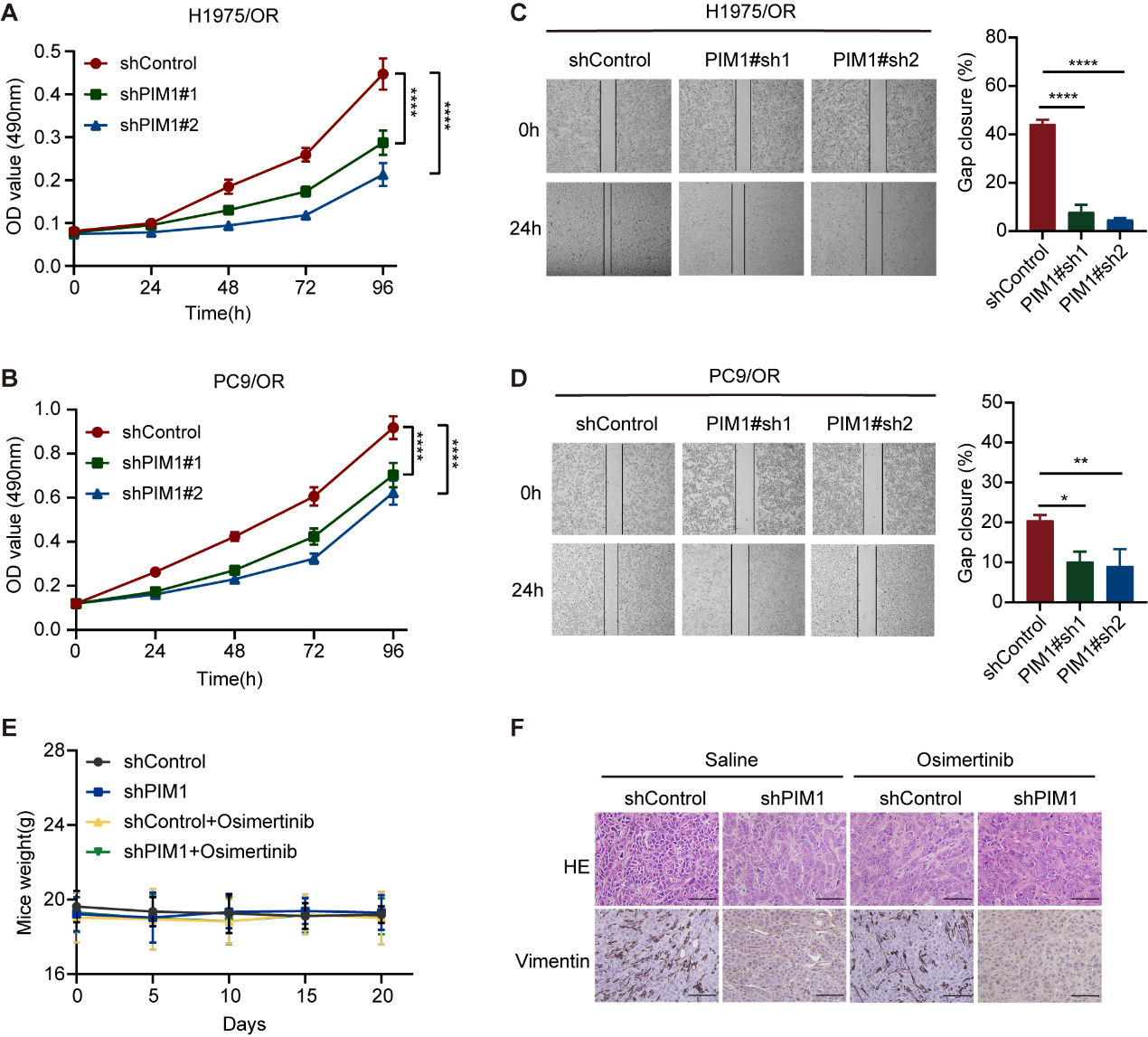
**

**Fig. S2.** (A, B) Proliferation curves of H1975/OR and PC9/OR cells after PIM1 knockdown. Data are presented as mean ± SD, ***p*<0.01, *****p*<0.0001, One-way ANOVA. (C, D) Knockdown of PIM1 inhibited motility capacity of H1975/OR and PC9/OR. Data are presented as mean ± SD, **p* < 0.05, ***p* < 0.01, *****p* < 0.0001, One-way ANOVA. (E) Measurement of mice weights. (F) H&E staining and Vimentin IHC staining of resected tumor tissues. Scale bar, 25 µm.


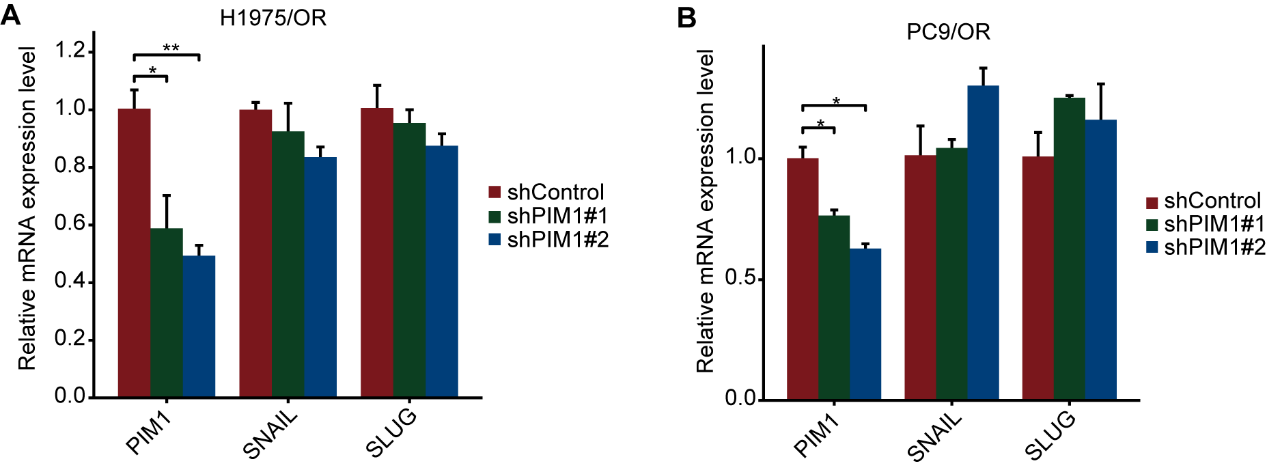


**Fig. S3.** (A) The mRNA expression levels of SNAIL and SLUG after PIM1 knockdown in H1975/OR cells. (B) The mRNA expression levels of SNAIL and SLUG after PIM1 knockdown in PC9/OR cells. PIM1 knockdown decreased SNAIL and SLUG protein expression levels, while mRNA expression levels were unchanged in H1975/OR and PC9/OR cells. Data are presented as mean ± SD, **p* < 0.05, ***p* < 0.01, Two-way ANOVA.
