# Supplementary material for: PIM1 kinase promotes EMT-associated osimertinib resistance via regulating GSK3β signaling pathway in EGFR-mutant non-small cell lung cancer
Source: Cell Death Dis. 2024 Sep 3;15(9):644. doi: 10.1038/s41419-024-07039-0 (PMC11372188; doi:10.1038/s41419-024-07039-0)
Supplement: Supplementary file 2 — Supplementary Tables [file 41419_2024_7039_MOESM2_ESM.docx]

**Supplementary Table S1. The primary antibodies utilized for immunoblotting, immunochemistry, and immunofluorescence** **analyses.**

| **Antibody** | **Source** | **Identifier** | **Dilution** |
| --- | --- | --- | --- |
| PIM1 | Abcam | ab54503 | 1:500 |
| PIM1 | Sigma | SAB1411969 | 1:50 |
| PIM1 | Abcam | ab75776 | 1:250 |
| SNAIL | Cell Signaling Technology | 3879 | 1:1000 |
| SLUG | Cell Signaling Technology | 9585 | 1:1000 |
| Vimentin | Cell Signaling Technology | 5741 | 1:1000 |
| Vimentin | Cell Signaling Technology | 5741 | 1:200 |
| Vimentin | Cell Signaling Technology | 5741 | 1:200 |
| E-cadherin | Cell Signaling Technology | 14472 | 1:1000 |
| GSK3β | Cell Signaling Technology | 12456 | 1:1000 |
| GSK3β | Cell Signaling Technology | 12456 | 1:400 |
| p-GSK3β (Ser9) | Cell Signaling Technology | 9323 | 1:1000 |
| p-GSK3β (Ser9) | Cell Signaling Technology | 9323 | 1:50 |
| p-GSK3β (Ser9) | Cell Signaling Technology | 9323 | 1:100 |
| p-GSK3β (Tyr216) | NOVUS | NB100-81946 | 1:1000 |
| GSK3α | Cell Signaling Technology | 4337 | 1:1000 |
| β-tubulin | Cell Signaling Technology | 2128 | 1:1000 |
| PIM1 | Santa Cruz | sc-13513 | 1:50 |
| Ubiquitin | Santa Cruz | sc-8017 | 1:1000 |
| Mouse IgG isotype control | Santa Cruz | sc-2025 | 1:200 |
| Rabbit IgG isotype control | Cell Signaling Technology | 3900 | 1:100 |

**Supplementary Table S2. shRNA sequences used in our research.**

| **shRNA** | **Sequences (5’-3’)** |
| --- | --- |
| shControl | TTCTCCGAACGTGTCACGT |
| shPIM1#1 | GGATCCTGCTGTATGATATGG |
| shPIM1#2 | GGCCAACCTTCGAAGAAATCC |

**Supplementary Table S3. siRNA sequences used in our research.**

| **siRNA** | **Sequences (5’-3’)** |
| --- | --- |
| siGSK3β | GGAAGCTTGTGCACATTCA |

**Supplementary Table S4. The primers used for real-time PCR in our research.**

| **Gene** | **Sequence** | |
| --- | --- | --- |
| PIM1 | Forward | TCCGCGTCTCCGACAACTT |
|  | Reverse | ACCTTCTTCAGCAGGACCACTT |
| SNAIL | Forward | GGAAGCCTAACTACAGCGAGCT |
|  | Reverse | CTGGAAGGTAAACTCTGGATTAG |
| SLUG | Forward | CTGTGACAAGGAATATGTGAGCC |
|  | Reverse | CAAATGCTCTGTTGCAGTGAG |
| GAPDH | Forward | GAAGGTGAAGGTCGGAG |
|  | Reverse | GAAGATGGTGATGGGATTTC |

**Supplementary Table S5. GO and KEGG results in our research.**

| **ONTOLOGY** | **ID** | **Description** | **p-adjust** |
| --- | --- | --- | --- |
| BP | GO:0050678 | regulation of epithelial cell proliferation | <0.001 |
| BP | GO:0048762 | mesenchymal cell differentiation | <0.001 |
| BP | GO:0007162 | negative regulation of cell adhesion | <0.001 |
| BP | GO:0071902 | positive regulation of protein serine/threonine kinase activity | 0.02 |
| BP | GO:0001837 | epithelial to mesenchymal transition | 0.05 |
| CC | GO:0062023 | collagen-containing extracellular matrix | <0.001 |
| CC | GO:0005911 | cell-cell junction | <0.001 |
| CC | GO:0030055 | cell-substrate junction | <0.001 |
| CC | GO:0005925 | focal adhesion | 0.01 |
| CC | GO:0005912 | adherens junction | 0.03 |
| MF | GO:0005201 | extracellular matrix structural constituent | <0.001 |
| MF | GO:0004714 | transmembrane receptor protein tyrosine kinase activity | <0.001 |
| MF | GO:0004713 | protein tyrosine kinase activity | <0.001 |
| MF | GO:0098632 | cell-cell adhesion mediator activity | 0.01 |
| MF | GO:0004674 | protein serine/threonine kinase activity | 0.02 |
| KEGG | hsa04512 | ECM-receptor interaction | <0.001 |
| KEGG | hsa04151 | PI3K-Akt signaling pathway | <0.001 |
| KEGG | hsa04514 | Cell adhesion molecules | 0.02 |
| KEGG | hsa04010 | MAPK signaling pathway | 0.02 |
| KEGG | hsa05222 | Small cell lung cancer | 0.06 |

**Supplementary Table S6. GSEA results in our research.**

| **MSigDB HALLMARK** | **Size** | **ES** | **NES** | **p-adjust** |
| --- | --- | --- | --- | --- |
| HALLMARK_EPITHELIAL_MESENCHYMAL_TRANSITION | 93 | 0.43 | 2.08 | <0.001 |
| HALLMARK_INFLAMMATORY_RESPONSE | 92 | 0.41 | 1.98 | <0.001 |
| HALLMARK_IL6_JAK_STAT3_SIGNALING | 34 | 0.51 | 1.98 | 0.01 |
| HALLMARK_TNFA_SIGNALING_VIA_NFKB | 95 | 0.36 | 1.77 | 0.01 |
| HALLMARK_ESTROGEN_RESPONSE_LATE | 92 | -0.37 | -1.65 | 0.03 |
| HALLMARK_APOPTOSIS | 51 | 0.36 | 1.55 | 0.18 |
