# Supplementary figures and images for: PIM1 kinase promotes EMT-associated osimertinib resistance via regulating GSK3β signaling pathway in EGFR-mutant non-small cell lung cancer
Source: Cell Death Dis. 2024 Sep 3;15(9):644. doi: 10.1038/s41419-024-07039-0 (PMC11372188; doi:10.1038/s41419-024-07039-0)

**Figure 1**

**
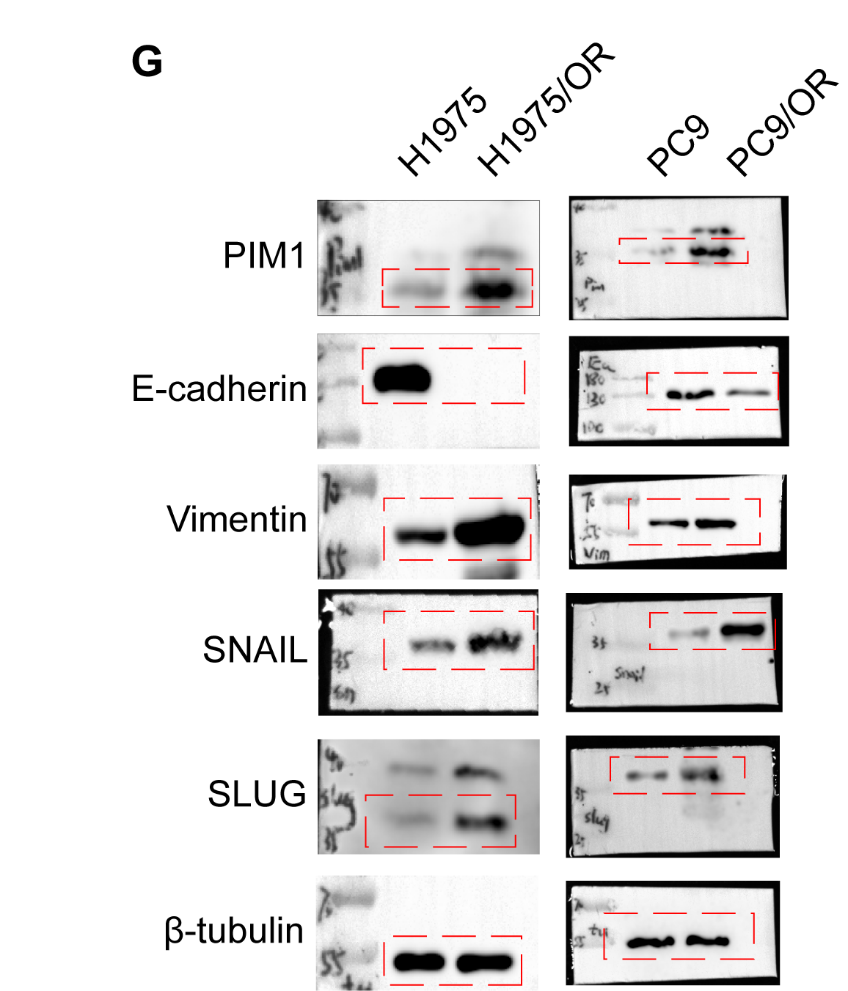
**

**Figure 2**

**
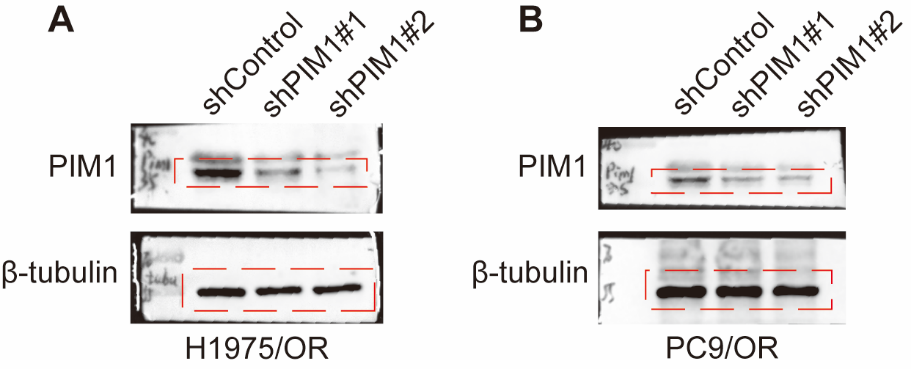
**

**Figure 3**

**
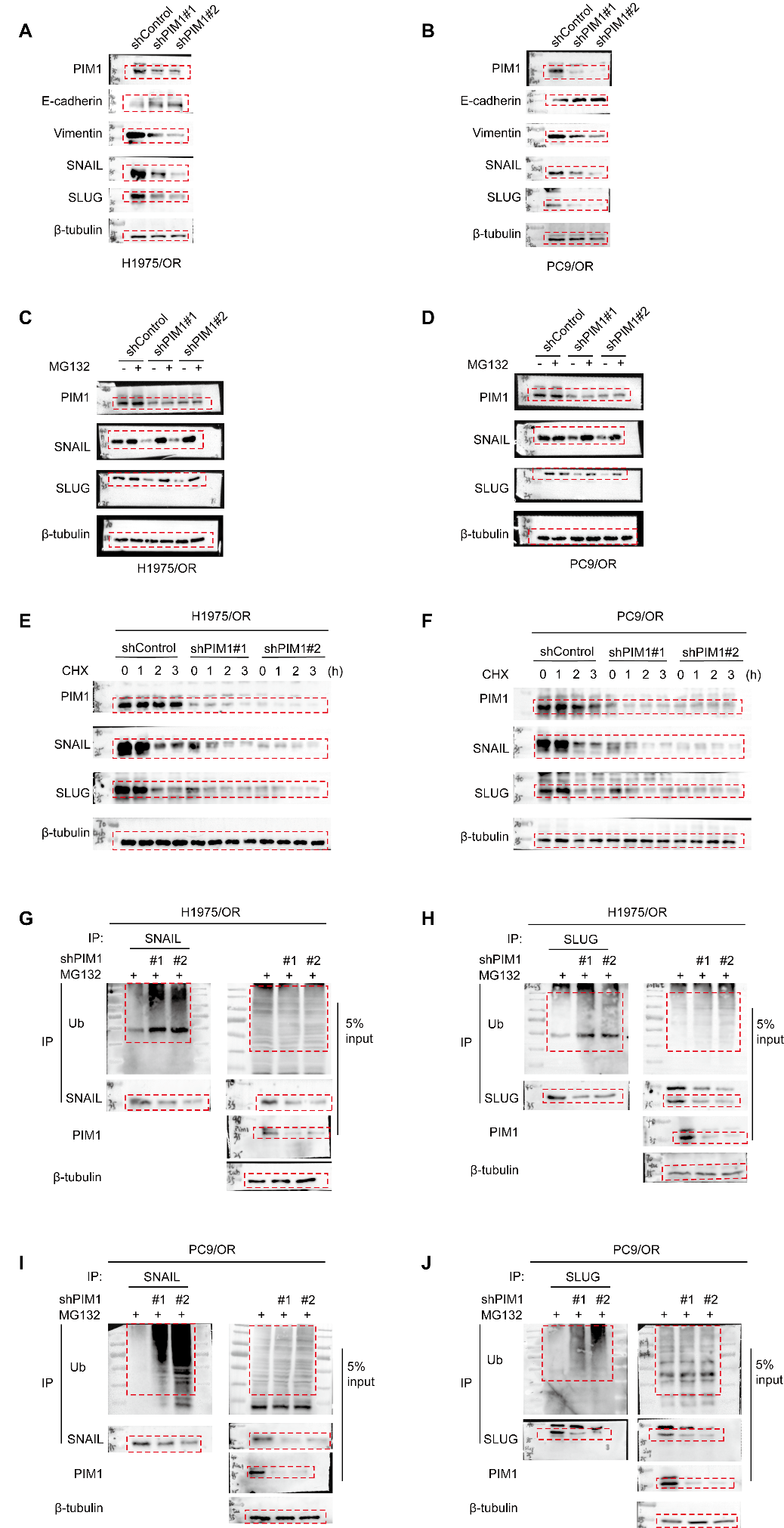
**

**Figure 4**

**
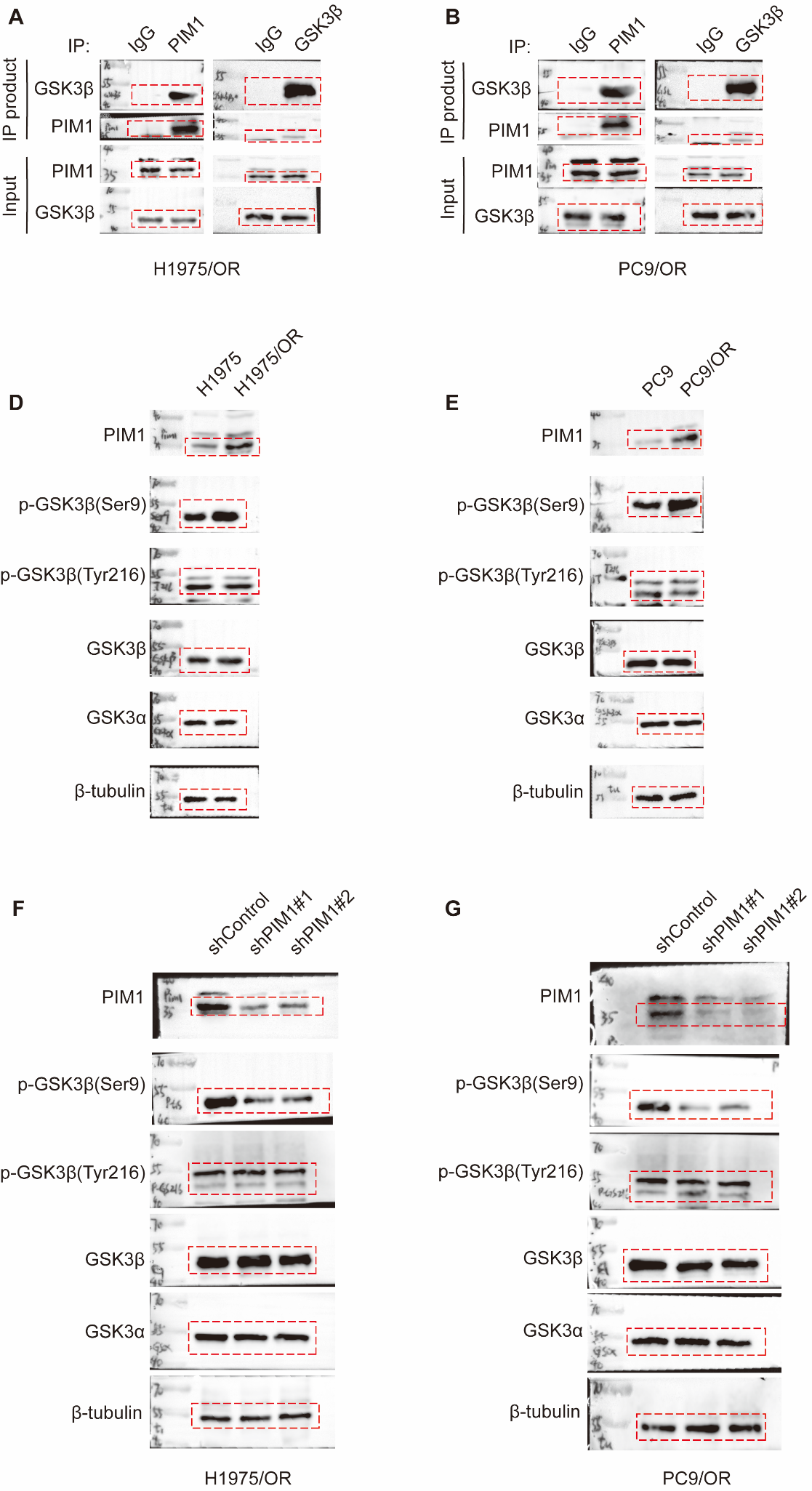
**

**Figure 5**

**
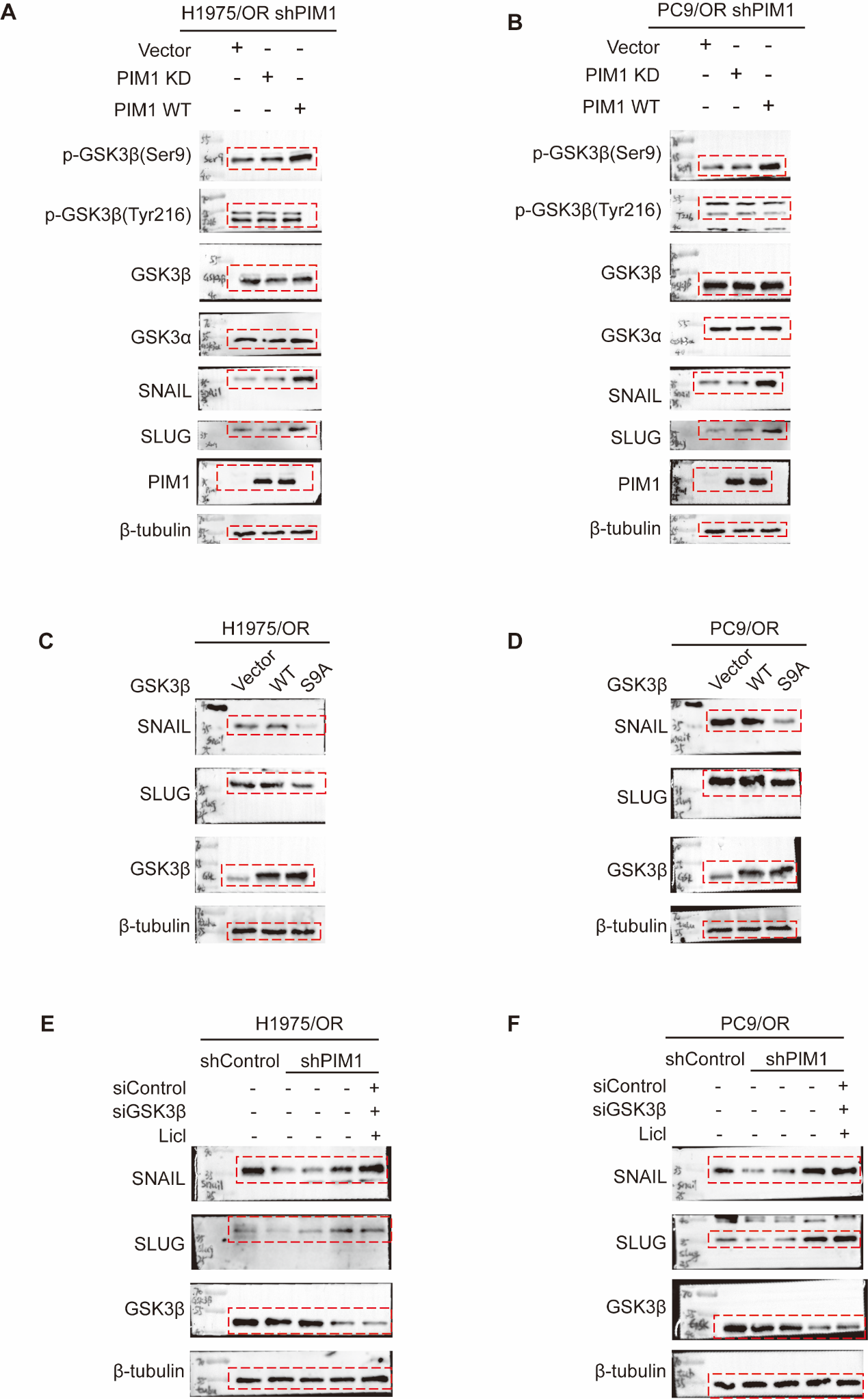
**

**Figure 6**

**
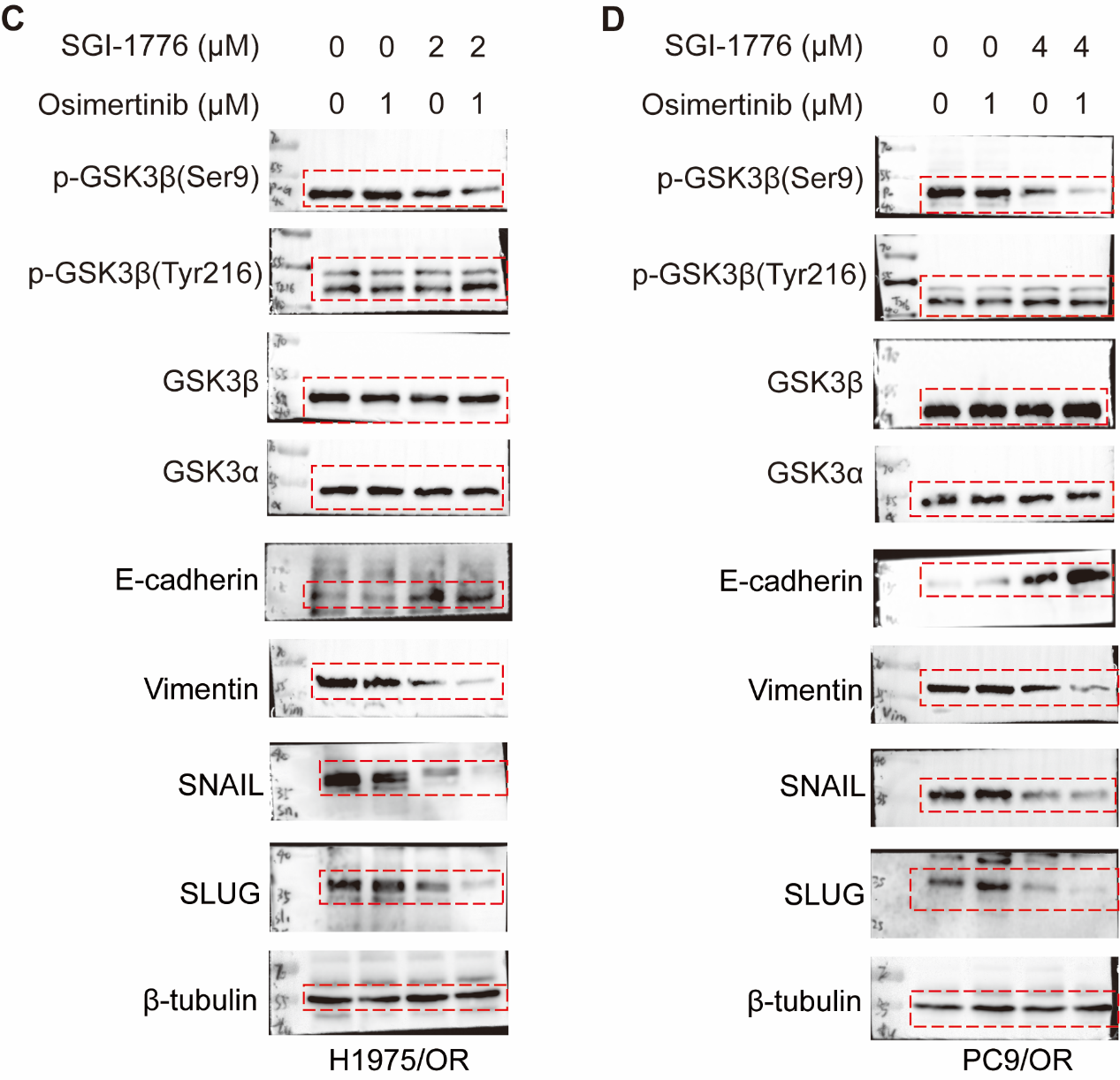
**

Supplement: Supplementary file 3 — Original western blots [file 41419_2024_7039_MOESM3_ESM.docx]
